# Adaptive and Dark Personality in the COVID-19 Pandemic: Predicting Health-Behavior Endorsement and the Appeal of Public-Health Messages

**DOI:** 10.1177/1948550620936439

**Published:** 2021-07

**Authors:** Pavel S. Blagov

**Affiliations:** 1 8233 Whitman College, Walla Walla, WA, USA

**Keywords:** communication, health, individual differences, personality

## Abstract

Who embraces directions to socially distance, boost hygiene, and protect others during a pandemic of contagious respiratory disease? Do differently phrased public-health messages appeal to different people? I based predictions on the five-factor, triarchic psychopathy, and Dark Triad models of normal-range and dark traits; the extended parallel process model (EPPM); and schema-congruence theory. In a survey of 502 online participants, normal-range traits (esp agreeableness and conscientiousness) predicted endorsement of social distancing and hygiene, as well as the appeal of health messages in general. Consistent with the EPPM, conscientiousness and neuroticism had an interaction. Dark traits (esp psychopathy, meanness, and disinhibition) predicted low endorsement of health behaviors and the intent to knowingly expose others to risk. Most participants preferred a message appealing to compassion (“Help protect the vulnerable…”), but dark traits predicted lower appeal of that message. Personality appears relevant to epidemiology and public-health communication in a contagious-disease context.

On March 11, 2020, the World Health Organization (WHO) announced a pandemic of *severe acute respiratory syndrome* (SARS) *coronavirus disease* (COVID-19). By March 20, all U.S. states had confirmed COVID-19 cases and announced states of emergency, to which people reacted in diverse ways (So et al., 2020). Were individual differences at play?

Personality predicts health and health behaviors broadly (Strickhouser et al., 2017), and disease avoidance may be one reason why traits like cautiousness, conformity, and social withdrawal have evolved (Lukaszewski & von Rueden, 2015; Schaller & Murray, 2008). Yet little is known about personality’s links to protective versus risk behavior germane to infectious disease, nor is there research on personality and responses to *public-health messages* (PHMs) in a pandemic. Who conforms to new hygiene and social distancing (SD) norms? What messages work for whom in helping contain a pandemic? I tested whether general, psychopathy-related, and dark personality dimensions correlated with people’s endorsement of key health behaviors. I also tested whether the congruence of PHMs with people’s traits predicted the messages’ appeal.

## Health Behavior and Normal-Range Personality

Early in the pandemic, authorities appealed to people’s responsibility (e.g., “wash your hands”), compassion and cooperation (e.g., “protect others, even if your risk is low”), and fear (e.g., by emphasizing COVID-19’s lethality). Such appeals do not presume individual differences yet call for behaviors that map readily onto the normal-range personality domains from the *five-factor model* (FFM; Digman, 1990).

One such domain, *conscientiousness*, predicts health-promoting and risk-avoiding behavior outside pandemic contexts (Roberts et al., 2005). *Agreeableness* (which entails empathy and cooperativeness) predicts compliance with social norms for specific health behaviors (e.g., in physical exercise, driving, sexual activity, alcohol use, and smoking; Malouff et al., 2006). *Neuroticism*, usually a health and risk behavior liability (Lahey, 2009), nevertheless may make people susceptible to “fear appeals” (dire warnings) to change behavior (Awagu & Basil, 2016). *Extroversion* likely makes SD aversive, and *openness* may boost health behaviors by improving perceptions of risk (Trobst et al., 2000). Overall, conscientiousness and agreeableness, the FFM’s prosocial domains, appear especially likely to predict health and risk behaviors.

More narrowly, personal hygiene’s links to personality have hardly been studied. Personality-bound processes like disease worrying (Liao et al., 2011) seemed to predict hygiene during an influenza pandemic, implicating neuroticism. Extroversion and (low) neuroticism may predict oral hygiene (Kressin et al., 1999).

Research on nonrespiratory communicable diseases and the FFM may inform predictions about responses to contagious SARS. Aspects of neuroticism, (low) conscientiousness, and (low) agreeableness may predict HIV risk behavior (Trobst et al., 2002) and sexually transmitted infection (Mõttus et al., 2012). Would this generalize to SARS?

Furthermore, conscientiousness and agreeableness may predict adaptive behavior in a pandemic synergistically, as they interact in academic and job performance (Witt et al., 2002). Additionally, the *extended parallel process model* (EPPM) of persuasion in PHMs suggests a synergy between threat sensitivity (entailed in neuroticism) and self-efficacy (linked to conscientiousness) in predicting people’s fear-appeal susceptibility (Witte, 1992).

## Health Behavior and Dark Personality

Apart from the FFM, clinical conceptualizations of maladaptive personality may explain people’s diverse reactions. Sensation seeking and neuroticism’s clinical manifestations predict many risk behaviors, including HIV-risk sexual behavior (Kalichman et al., 1998; Razei et al., 2017), so do antisocial and borderline personality disorder features (Adams et al., 2016; Kelley & Petry, 2000; Ladd & Petry, 2003). Because it relates to these disorders and sensation seeking, such findings implicate psychopathy.

*Psychopathy* is a cluster of characteristics revolving around unempathic callousness (Verschuere et al., 2018) and including egocentricity, grandiosity, glibness, remorselessness, deceptiveness, manipulativeness, recklessness, unreliability, and antisociality (Hare, 1996). It partially overlaps with the other *Dark Triad* traits (Paulhus & Williams, 2002): *narcissism* (which involves more exhibitionism and less disinhibition) and *Machiavellianism* (which entails more cynicism and calculated exploitativeness and less grandiosity and disinhibition). Not unitary, in Patrick et al.’s (2009) triarchic model, psychopathy is the confluence of *boldness* (dominance, fearlessness, adventurousness, and stress immunity), *disinhibition* (lack of constraint, especially of one’s antisocial impulses), and *meanness* (callousness and instrumental exploitativeness).

Psychopathy and, to some extent, narcissism and Machiavellianism predict not only antisocial tendencies (including aggression and violence) but also various health behaviors and outcomes (Hudek-Knezevic et al., 2016; Jonason et al., 2015; Malesza & Kaczmarek, 2019), including HIV-risk sexual behavior (Hudek-Knezevic et al., 2007). Like the FFM’s normal-range traits, the Dark Triad has not been studied in relation to PHMs or health-related behaviors in a SARS pandemic.

## Dark Personality and Harm to Others’ Health

If maladaptive personality partially explains reactions to the pandemic and to PHMs, it matters to public health. For example, it may explain who intentionally spreads, or threatens to spread, disease—which raises societal concern (Dryer, 2020). Sensation seeking’s link to HIV-risk behavior in infected persons has such implications (Kalichman et al., 2008; Shuper et al., 2014). Findings link psychopathy to deliberately misleading sex partners about one’s HIV-positive status (Benotsch et al., 2012) and narcissism to knowingly putting others at risk of HIV (Martin et al., 2013). More generally, psychopathy and the Dark Triad correlate with endorsement of unethical behavior (Pletti et al., 2017; Roeser et al., 2016). During a pandemic, dark personality may predispose people to dismiss or act contrary to PHMs and endanger others.

## Personality and Health Communications

Above, I theorized links between personality and health-related behavior during the COVID-19 pandemic. Additionally, personality may have applied implications (Ferguson, 2013), particularly for communicating health advice (Dutta-Bergman, 2003). It may partly explain what PHMs are persuasive to different people. According to *schema-congruence theory* (Brock et al., 1990), people prefer and find more persuasive messages that align with their views of themselves. This has been suggested in commercial advertising (Chang, 2005; Matz et al., 2017), antismoking campaigns (Chang, 2009), diabetes education (Lawson et al., 2010), college drinking (York et al., 2012a), dental-hygiene promotion (Sherman et al., 2008), and condom use (Noar et al., 2006). If personality is reflected in their self-concept, people may favor messages that match their self-reported traits.

## The Current Study

Given the paucity of research on personality in relation to SARS-protective and risk behaviors during a pandemic, I tested such relationships using a survey. Measuring actual behaviors would be preferable, yet self-reported current and intended behaviors provide a starting point to probe the boundaries of personality theory.

Among the constructs discussed above, conscientiousness, agreeableness, (low) extroversion, neuroticism, (low) boldness, and (low) disinhibition emerged as candidates to predict self-reported ongoing SD. Conscientiousness, agreeableness, neuroticism, low boldness, disinhibition, and psychopathy may predict endorsement of intent for future SD (presumably because of growing knowledge and PHMs about the pandemic). Conscientiousness, agreeableness, (low) disinhibition, and (low) meanness are likely to predict intent for future hygiene (to protect not only the self but also others). Psychopathy, meanness, disinhibition, and Machiavellianism may predict endorsing the willingness to infect others.

Additionally, conscientiousness and agreeableness, and conscientiousness and neuroticism would interact in predicting current SD and future intent for SD (as per prior research cited above and the EPPM).

Per schema-congruence theory, as outlined above, differently phrased PHMs’ appeal would correlate with personality dimensions: (a) for a “self-centered” message, with narcissism, meanness, Machiavellianism, and psychopathy; (b) for a “responsible” message, with conscientiousness and (low) disinhibition; (c) for a “compassionate” message, with agreeableness, conscientiousness, (low) narcissism, (low) Machiavellianism, (low) psychopathy, and (low) meanness; (d) for an “avoidant” message, with neuroticism and (low) boldness; and (e) for a “sociable” message, with extroversion, boldness, and openness.

## Method

After preregistering the study on March 20, I collected data from March 20 to 23, just after California and New York (followed by other states) issued shelter-at-home orders. At that time, Americans had varied and evolving opinions about the pandemic, whether it posed risk to them and whether the public was overreacting (Rakich, 2020).

### Participants

U.S. Mechanical Turk (MTurk) workers with master status (*N* = 502) received US$2.50 to complete a 15-min survey. Expecting small effect sizes and limited by funding, I planned to obtain 500 cases for .85 power (Algina & Olejnik, 2003) to detect partial correlations of .15 at *p* = .01 (one-tailed) after data cleaning. I anticipated careless (possibly automated) responding on MTurk and planned to screen the data for duplicate, automated, and invalid responding before testing hypotheses. Of 652 people who reached the survey’s end, 616 passed effort checks. Of them, 562 passed validity checks, 540 passed subsequent inconsistency checks, 503 passed a completion-time check, and 502 had fully completed all questionnaires (Supplemental Material Tables S1–S4). No duplicates were evident. Thus, 23% of response sets were excluded as noncompliant or suspicious. Participants reported being 51% male, 77.5% White, 47.2% single, and 60.4% full-time employed. Age ranged from 21 to 76 years (*M* = 41, *SD* = 11), and 72.5% denied having health conditions that increase risk from COVID-19 (Table S8).

### Measures

Based on WHO and Centers for Disease Control and Prevention advice for preventing COVID-19’s spread, I listed 10 health behaviors coinciding with widely popularized advice (Nierenberg, 2020). Conceptually, they represent SD and *hygiene*. From that list, I wrote two measures to capture self-reported current and intended future health behaviors (FHBs). A third questionnaire, with broader content, was intended to measure covertly participants’ willingness to deliberately put others at risk (vs. following advice to protect others).

Current health behavior (CHB; 10 items) asked participants how much they currently did each behavior as a precaution against COVID-19 (e.g., “Limit contact with people as much as possible…”) on a 4-point scale from *not at all or never* to *extremely or always*. I expected the data to be reducible to *CHB: SD* and *CHB: hygiene*. A principal component analysis (PCA) corroborated this (Table S5). Thus, although CHB’s internal consistency was high, α = .89, I reduced its data to two dimensions. Table S10 contains descriptives.

FHB (14 items) asked participants to rate on a 5-point scale from *extremely likely* to *not likely at all* their likelihood to do each behavior over the next 2 weeks. Scales were keyed opposite to CHBs (and later reversed) for data control purposes. Ten items paralleled the CHBs. Four captured more venturous interpersonal contact, from going to a large gathering to having sex with someone new. I expected the data to be reducible to *FHB: SD, FHB: hygiene,* and *venturous behavior*. Internal consistency was acceptable, α = .76, and PCA supported the intended structure (Table S6).

Carrier scenario (CS; 18 items) asked participants to rate on a 4-point scale (from *not at all* to *definitely*) the likelihood of specific behaviors if they had good reason to think they were carrying COVID-19. The behaviors ranged from calling ahead prior to seeing a physician, to wearing a mask, to shaking hands in different situations, and to purposefully trying to infect others. CS (α = 71) was readily reducible to two components (Table S7): *harmful behavior* (HB) and *protecting others* (POs).

#### PHM appeal

Participants viewed, in a randomized order, five PHMs written to appeal to different personalities: self-centered, responsible, compassionate, avoidant, and sociable (Appendix SA). They rated from 0 to 100 how much each message (a) was persuasive to them, (b) was likely for them to take seriously, and (c) was likely to affect their behavior. Each PHM’s composite score was internally consistent (α = .93 to .95). Participants also viewed distinctive lines from each message with instructions to rank order the PHMs based on personal appeal.

#### Personality dimensions

The Mini-International Personality Item Pool (IPIP) (MIP; Donnellan et al., 2006) is 20-item form of the IPIP (Goldberg, 1992, 1999) developed to maximize short-form validity and factor separation. It captures the FFM’s general trait domains (agreeableness, conscientiousness, etc.). Participants responded on a 5-point scale from *very false* to *very true*. For exploratory purposes, I computed personality superfactors: stability, plasticity, and the general *P* factor (Rushton & Irwing, 2011; Tables S11 and S15).

The Abbreviated Measure of Psychopathy (AMP; Semel, 2018) operationalizes the triarchic psychopathy model with 33 items as a parsimonious alternative to Patrick’s (2010) longer instrument. It has a 4-point scale from *true* to *false* and yields scores on boldness (e.g., “I have a very strong and dominating personality”), meanness (e.g., “It sometimes gives me pleasure to see someone in pain”), and disinhibition (e.g., “The saying ‘plan ahead’ is definitely not for me”). For exploratory purposes, I also employed the overall score.

The Short Dark Triad (SD3; Jones & Paulhus, 2014) is a 27-item measure yielding scores on narcissism (e.g., “Many group activities tend to be dull without me”), Machiavellianism (e.g., “I like to use clever manipulation to get my way”), and psychopathy (e.g., “Payback needs to be quick and nasty”). Participants responded on a 5-point scale from *disagree strongly* to *agree strongly*. I also utilized its overall score to estimate the dark factor, *D* (Moshagen et al., 2020).

#### Additional considerations

To minimize the effects of global response sets, I keyed the measures’ scales both ways. Additionally, about half of the MIP and multiple AMP and SD3 items were reverse keyed. Personality questionnaire names were disguised.

I planned to control for gender, age, and presence of health conditions that increase risk from COVID-19. These could be expected to covary with both personality and pandemic-related behavior. Extreme responses are of interest when studying maladaptive behavior, so I did not plan deleting outliers. I adopted α = .01 (one-tailed) for 37 partial-correlation hypotheses (as noted earlier) and α = .05 for four interaction hypotheses_._

## Results

### CHB

As predicted, agreeableness (*r_p_* = .21, *p* = 2.6^−6^), conscientiousness (*r_p_* = .18, *p* = 6.3^−5^), and neuroticism (*r_p_* = .12, *p* = 8.4^−3^) predicted CHB: SD (Table 1 includes confidence intervals [CIs]). In *exploratory analyses* (*EAs*), so did extroversion (*r_p_* = .12, *p* = 6.1^−3^) and the FFM superfactors, especially stability (*r_p_* = .24, *p* = 4.3^−8^; Table S16).

**Table 1.** Partial Correlations Between Health Behavior Endorsement and Normal Personality Domains.

Mini-IPIP Factors and Superfactors		Current Health Behavior				Future Health Behavior						Carrier Scenario			
		Social Distancing		Hygiene		Social Distancing		Hygiene		Venturous Behavior		Harmful Behavior		Protecting Others	
Agreeableness	*r_p_/p*	.21^†^	2.6E-06	.32^†^	3.1E-13	.16^†^	2.4E-04	.32^†^	2.5E-13	−.01	4.3E-01	−.17^†^	1.1E-04	.15^†^	2.7E-04
98% b.c. CI		[.07, .34]		[.20, .43]		[.02, .30]		[.19, .44]		[−.14, .10]		[−.28, −.03]		[.05, .26]	
Conscientiousness	*r_p_/p*	.18^†^	6.3E-05	.25^†^	1.2E-08	.15^†^	2.6E-04	.24^†^	4.0E-08	−.12*	2.7E-03	-.07	7.2E-02	.12*	3.0E-03
98% b.c. CI		[.07, .28]		[.13, .37]		[.03, .29]		[.13, .35]		[−.27, .02]		[−.15, .01]		[.03, .22]	
Neuroticism	*r_p_/p*	.12*	8.4E-03	.22^†^	6.3E-07	.07	5.7E-02	.18^†^	1.9E-05	.01	3.9E-01	−.05	1.2E-01	.15^†^	3.8E-04
98% b.c. CI		[.01, .23]		[.11, .32]		[−.03, .18]		[.08, .30]		[−.08, .11]		[−.17, .05]		[.04, .25]	
Extroversion	*r_p_/p*	−.12*	6.1E-03	−.22^†^	4.5E-07	−.12*	3.1E-03	−.22^†^	5.1E-07	.06	8.1E-02	.09	2.6E-02	−.10	1.1E-02
98% b.c. CI		[−.23, −.03]		[−.31, −.13]		[−.25, −.01]		[−.33, −.12]		[−.05, .17]		[.00, .22]		[−.21, .01]	
Openness	*r_p_/p*	.10	2.8E-02	.15^†^	5.4E-04	−.01	4.6E-01	.09	2.7E-02	.02	3.6E-01	−.08	4.4E-02	.09	2.5E-02
98% b.c. CI		[.00, .20]		[.05, .25]		[−.10, .09]		[−.02, .20]		[−.11, .12]		[−.24, .10]		[−.02, .20]	

*Note*. *N* = 502. Controlling for sex, age, and risk health condition. Shaded: Tests with a priori hypotheses. Unshaded: Exploratory analyses. Bootstrap estimation of b.c. CI with 1,000 iterations. b.c. CI = bias corrected confidence interval.

*p < .01 = 1.0^−2^. ^†^ *p* < .001 = 1.0^−3^.

Contrary to prediction, boldness did not predict CHB: SD (*r_p_* = −.03, *p* = 5.9^−1^; Table 2). As hypothesized, disinhibition (*r_p_* = −.17, *p* = 9.5^−5^) and SD3 psychopathy did (*r_p_* = −.23, *p* = 1.2^−7^). In EAs, so did meanness (*r_p_* = −.17, *p* = 1.2^−4^), Machiavellianism (*r_p_* = −.16, *p* = 3.1^−4^), overall AMP psychopathy (*r_p_* = −.15, *p* = 9.2^−4^), and SD3 dark factor (*D*; *r_p_* = −.14, *p* = 1.5^−3^) scores.

**Table 2.** Partial Correlations Between Health Behavior Endorsement and Dark Personality Traits.

Dark Personality Dimensions		Current Health Behavior				Future Health Behavior						Carrier Scenario			
		Social Distancing		Hygiene		Social Distancing		Hygiene		Venturous Behavior		Harmful Behavior		Protecting Others	
AMP boldness	*r_p_/p*	−.03	5.0E-01	.04	3.3E-01	−.09	2.5E-02	.01	4.9E-01	.11*	7.5E-03	.05	1.5E-01	−.04	1.7E-01
98% b.c. CI		[−.13, .07]		[−.07, .16]		[−.21, .05]		[−.13, .13]		[.00, .22]		[−.08, .16]		[−.15, .07]	
AMP meanness	*r_p_/p*	−.17^†^	1.3E-04	−.24^†^	5.5E-08	−.20^†^	2.4E-06	−.28^†^	1.6E-10	.13*	2.5E-03	.21^†^	2.0E-06	−.21^†^	1.9E-06
98% b.c. CI		[−.29, −.05]		[−.36, −.11]		[−.35, −.07]		[−.41, −.14]		[−.02, .29]		[.07, .31]		[−.31, −.11]	
AMP disinhibition	*r_p_/p*	−.17^†^	9.5E-05	−.21^†^	2.8E-06	−.23^†^	1.2E-07	−.25^†^	1.2E-08	.21^†^	1.2E-06	.19^†^	1.3E-05	−.19^†^	6.8E-06
98% b.c. CI		[−.28, −.07]		[−.32, −.07]		[−.37, −.08]		[−.38, −.12]		[.05, .36]		[.03, .31]		[−.29, −.09]	
SD3 narcissism	*r_p_/p*	.03	5.6E-01	.08	6.5E-02	.00	4.6E-01	.06	9.7E-02	.03	2.2E-01	.05	1.4E-01	.02	3.2E-01
98% b.c. CI		[−.08, .13]		[−.03, .20]		[−.11, .12]		[−.06, .18]		[−.07, .16]		[−.06, .14]		[−.09, .13]	
SD3 Machiavellianism	*r_p_/p*	−.16^†^	3.1E-04	−.19^†^	1.9E-05	−.13*	2.6E-03	−.17^†^	5.1E-05	.08	4.6E-02	.10	1.1E-02	−.17^†^	1.0E-04
98% b.c. CI		[−.26, −.06]		[−.30, −.08]		[−.26, −.01]		[−.29, −.06]		[−.06, .23]		[.03, .21]		[−.26, −.07]	
SD3 psychopathy	*r_p_/p*	−.23^†^	1.2E-07	−.20^†^	7.0E-06	−.20^†^	2.3E-06	−.21^†^	1.1E-06	.18^†^	4.2E-05	.18^†^	2.9E-05	−.10	1.1E-02
98% b.c. CI		[−.34, −.12]		[−.31, −.07]		[−.34, −.07]		[−.33, −.08]		[.01, .33]		[.05, .28]		[−.21, .00]	

*Note. N* = 502. Controlling for sex, age, and risk health condition. Shaded: Tests with a priori hypotheses. Unshaded: Exploratory analyses. Bayesian estimation of b.c. CI with 1,000 iterations. AMP = Abbreviated Measure of Psychopathy; SD3 = Short Dark Triad; b.c. CI = bias corrected confidence interval.

*p < .01 = 1.0^−2^. ^†^ *p* < .001 = 1.0^−3^.

### FHB

As predicted, conscientiousness correlated with FHB: SD (*r_p_* = .15, *p* = 2.6^−4^). Contrary to predictions, neuroticism did not (*r_p_* = .07, *p* = 5.7^−2^). In EAs, agreeableness (*r_p_* = .16, *p* = 2.4^−4^) and, negatively, extroversion (*r_p_* = −.12, *p* = 3.1^−3^) were linked to FHB: SD. As with CHB: SD, the superfactors stability (*r_p_* = .21, *p* = 1.6^−6^) and *P* (*r_p_* = .16, *p* = 2.4^−4^) predicted FHB: SD.

As predicted, disinhibition (*r_p_* = −.23, *p* = 1.2^−76^) and SD3 psychopathy (*r_p_* = −.20, *p* = 2.3^−6^) correlated inversely with FHB: SD. Contrary to the hypothesis, boldness did not (*r_p_* = −.09, *p* = 2.5^−2^). In EAs, meanness (*r_p_* = −.20, *p* = 2.4^−6^), Machiavellianism (*r_p_* = −.13, *p* = 2.6^−3^), AMP psychopathy (*r_p_* = −.21, *p* = 1.3^−6^), and SD3: *D* (*r_p_* = −.13, *p* = 2.5^−3^) predicted lower FHB: SD.

Regarding FHB: hygiene (H), the hypotheses for agreeableness (*r_p_* = .32, *p* = 2.5^−13^) and conscientiousness (*r_p_* = .24, *p* = 4.0^−8^) received support. In EAs, neuroticism (*r_p_* = .18, *p* = 1.9^−5^) and, negatively, extroversion (*r_p_* = −.22, *p* = 5.1^−7^) correlated with FHB: H, as did stability (*r_p_* = .37, *p* = 6.9^−18^) and *P* (*r_p_* = .33, *p* = 2.9^−14^).

Also supported were the predictions that meanness (*r_p_* = −.28, *p* = 1.6^−10^) and disinhibition (*r_p_* = −.25, *p* = 2.6^−8^) would predict FHB: H negatively. In EAs, so did Machiavellianism (*r_p_* = −.17, *p* = 5.1^−5^) and psychopathy (*r_p_* = −.21, *p* = 1.1^−6^), as did overall AMP psychopathy (*r_p_* = −.20, *p* = 2.2^−6^) and SD3: *D* (*r_p_* = −.11, *p* = 2.8^−3^).

In EAs, FHB: venturous behavior correlated especially with disinhibition (*r_p_* = .21, *p* = 1.2^−6^) and, more generally, overall AMP psychopathy (*r_p_* = .18, *p* = 2.6^−5^).

### CS: HB

As predicted, meanness (*r_p_* = .21, *p* = 2.0^−6^) and disinhibition (*r_p_* = .19, *p* = 1.3^−5^), and SD3 psychopathy (*r_p_* = .18, *p* = 2.9^−5^), predicted CS: HB. The result for Machiavellianism (*r_p_* = .10, *p* = 1.1^−2^) approached α = .01 (one-tailed) but was nonsignificant; however, its 98% CI excluded 0, suggesting that it, too, predicted CS: HB. In EAs, so did overall AMP psychopathy (*r_p_* = .17, *p* = 4.4^−5^) and SD3: *D* (*r_p_* = .13, *p* = 1.5^−3^), as did agreeableness (negatively; *r_p_* = −.17, *p* = 1.1^−4^). EAs suggested that CS: POs was negatively linked especially to meanness (*r_p_* = −.21, *p* = 1.9^−6^), disinhibition (*r_p_* = −.19, *p* = 6.8^−6^), and Machiavellianism (*r_p_* = −.17, *p* = 1.0^−4^).

### Interactions

As predicted, conscientiousness and neuroticism interacted (β = −.11, *p* = 8.0^−3^; Table 3) in predicting CHB: SD (after controlling for age, gender, and health risk). Specifically, CHB: SD (corrected for age, gender, and risk) was less correlated with neuroticism (β = −.01) at lower conscientiousness levels than at higher ones (*M* = 3.6, *SD* = 0.78, β = −.20). The hypothesis that conscientiousness and neuroticism would interact in predicting FHB: SD (β = −.06, *p* = 2.0^−1^) was not supported.

**Table 3.** Tests of Interactions Between Conscientiousness and Agreeableness, and Conscientiousness and Neuroticism, in Predicting Social Distancing Endorsement.

Model	Step 2	Predictors	*R*	Adjusted *R*	*p*	β	*p*	*b*	95% b.c. CI	VIF	DW
1	Current social distancing	Conscientiousness (C)	.35^†^	.111^†^	5.8E-12	.14^†^	1.2E-3	.08	[0.03, 0.04]	1.04	2.03
		Agreeableness (A)				.18^†^	6.1E-5	.10	[0.04, 0.16]	1.11	
		Interaction: C × A				−.05	2.9E-1	−.02	[−0.09, 0.03]	1.02	
2	Current social distancing	Conscientiousness (C)	.33^†^	.096^†^	2.7E-10	.16^†^	5.1E-4	.09	[0.03, 0.15]	1.11	2.08
		Neuroticism (N)				−.08	6.6E-2	−.05	[−0.11, 0.01]	1.17	
		Interaction: C × N				−.11^†^	8.0E-3	−.06	[−0.11, −0.01]	1.01	
3	Future social distancing	Conscientiousness (C)	.31^†^	.085^†^	4.2E-9	.12^†^	4.5E-3	.10	[0.01, 0.18]	1.04	1.99
		Agreeableness (A)				.13^†^	5.5E-3	.10	[0.02, 0.20]	1.11	
		Interaction: C × A				−.09*	2.9E-2	−.07	[−0.15, 0.04]	1.02	
4	Future social distancing	Conscientiousness (C)	.28^†^	.070^†^	2.1E-7	.13^†^	4.3E-3	.11	[0.01, 0.21]	1.11	2.08
		Neuroticism (N)				−.09	5.7E-2	−.07	[−0.16, 0.01]	1.17	
		Interaction: C × N				−.06	2.0E-1	−.04	[−0.16, 0.07]	1.01	

*Note. N* = 502. Each model controls for age, gender identity, and health risk status in the previous step omitted for brevity. All predictors were centered. Bootstrap estimation of b.c. CI based on 1,000 iterations. Shaded are tests of hypothesized interactions. VIF = variance inflation factors; DF = Durbin–Watson statistic.

*p < .05 = 5.0^−2^. ^†^ *p* < .01 = 1.0^−3^.

The hypothesis that conscientiousness and agreeableness interact to predict FHB: SD received support at α = .05 (β = .09, *p* = 2.9^−2^), but the 95% CI for *b* included 0, rendering this inconclusive. It raises the possibility that conscientiousness and agreeableness may predict FHB: SD synergistically, conscientiousness predicting FHB: SD slightly less well at higher (β = .12, *p* = 7.4^−2^) than lower (β = .17, *p* = 4.3^−3^) agreeableness levels. The hypothesis that conscientiousness and agreeableness would interact in predicting CHB: SD did not receive support (β = −.05, *p* = 2.9^−1^).

### PHMs

All PHMs received high mean ratings (Table S10). The mean appeal of the compassionate (highest, *M* = 84, *SD* = 19.4) and sociable (lowest, *M =* 66, *SD* = 28.7) messages differed from the rest (Figure S1), *F*(1, 501) = 102, *p* < 2.73^−79^, partial η^2^ = .17 (large effect).

The hypotheses that PHM1: self-centered’s appeal would correlate *positively* with meanness (*r_p_* = −.14, *p* = 1.2^−3^), narcissism (*r_p_* = .08, *p* = 3.6^−2^), Machiavellianism (*r_p_* = −.11, *p* = 5.5^−3^), and SD3 psychopathy (*r_p_* = −.14, *p* = 1.1^−3^) did not receive support. These associations were mostly *negative.* Adaptive traits, for example, the superfactors stability (*r_p_* = .27, *p* = 7.1^−10^), plasticity (*r_p_* = .17, *p* = 9.7^−5^), and *P* (*r_p_* = .26, *p* = 2.2^−9^) predicted this and all other PHMs’ appeal (Tables 4 and S17**)**.

**Table 4.** Partial Correlations Between Public-Health Message Appeal and Personality Traits.

Personality Dimensions		Public-Health Message Appeal									
		Self-Centered		Responsible		Compassionate		Avoidant		Sociable	
MIP A	*r_p_/p*	0.26^†^	2.7E-09	.29^†^	3.6E-11	.37^†^	5.6E-18	.24^†^	2.7E-08	.21^†^	6.5E-07
98% b.c. CI		[0.13, .38]		[.16, .41]		[.26, .48]		[.11, .37]		[.09, .35]	
MIP C	*r_p_/p*	0.18	3.1E-05	.15^†^	4.6E-04	.17^†^	8.0E-05	.14^†^	6.2E-04	.09	2.5E-02
98% b.c. CI		[0.08, .28]		[.04, .25]		[.06, .27]		[.02, .25]		[−.03, .20]	
MIP N	*r_p_/p*	0.16^†^	1.7E-04	.16^†^	1.3E-04	.11*	9.5E-03	.13*	2.2E-03	.18^†^	2.1E-05
98% b.c. CI		[0.05, .26]		[.06, .26]		[.00, .21]		[.02, .23]		[.07, .28]	
MIP E	*r_p_/p*	−0.13*	2.6E-03	−.11*	6.4E-03	−.11*	6.0E-03	−.09	2.2E-02	−.10	1.4E-02
98% b.c. CI		[−0.23, −.02]		[−.22, −.01]		[−.23, .01]		[−.20, .02]		[−.22, .01]	
MIP O	*r_p_/p*	0.10	1.1E-02	.11*	6.1E-03	.15^†^	3.3E-04	.09	2.1E-02	.06	8.2E-02
98% b.c. CI		[−0.01, .20]		[.00, .22]		[.05, .25]		[−.02, .20]		[−.05, .17]	
AMP B	*r_p_/p*	0.02	3.1E-01	.02	3.4E-01	−.07	5.6E-02	.01	4.6E-01	.03	2.6E-01
98% b.c. CI		[−0.08, .13]		[−.09, .14]		[−.18, .04]		[−.10, .09]		[−.08, .14]	
AMP M	*r_p_/p*	−0.14*	1.2E-03	−.19^†^	1.3E-05	−.31^†^	3.3E-13	−.11*	8.3E-03	−.13*	2.3E-03
98% b.c. CI		[−0.25, −.02]		[−.31, −.06]		[−.42, −.19]		[−.22, .00]		[−.23, −.03]	
AMP D	*r_p_/p*	−0.10*	1.0E-02	−.11*	7.1E-03	−.24^†^	1.7E-08	−.10	1.6E-02	−.06	1.1E-01
98% b.c. CI		[−0.20, .00]		[−.21, −.01]		[−.35, −.13]		[−.20, .00]		[−.15, .03]	
SD3 N	*r_p_/p*	0.08	3.6E-02	.08	3.4E-02	−.04	2.0E-01	.09	2.7E-02	.12*	4.8E-03
98% b.c. CI		[−0.03, .18]		[−.03, .18]		[−.14, .06]		[−.01, .18]		[.00, .22]	
SD3 Mch	*r_p_*/*p*	−0.11*	5.5E-03	−.17^†^	8.9E-05	−.23^†^	1.6E-07	−.03	2.7E-01	−.05	1.4E-01
98% b.c. CI		[−0.22, .00]		[−.26, −.06]		[−.32, −.13]		[−.13, .07]		[−.15, .06]	
SD3 P	*r_p_/p*	−0.14*	1.1E-03	−.14^†^	7.3E-04	−.26^†^	2.5E-09	−.12*	4.8E-03	−.08	3.5E-02
98% b.c. CI		[−0.25, −.03]		[−.26, −.04]		[−.36, −.15]		[−.22, −.02]		[−.18, .02]	

*Note. N* = 502. Controlling for sex, age, and risk health condition. Shaded: tests with a priori hypotheses. Unshaded: exploratory. Bootstrapping of b.c. CI with 1,000 iterations. MIP = mini-IPIP (A = agreeableness, C = conscientiousness, N = neuroticism, E = extroversion, and O = openness); AMP = Abbreviated Measure of Psychopathy (B = boldness, M = Machiavellianism, and D = disinhibition); SD3 = Short Dark Triad (N = narcissism, Mch = Machiavellianism, and P = psychopathy).

*p < .01 = 1.0^−2^. ^†^ *p* < .001 = 1.0^−3^.

The hypotheses that PHM2: Responsible’s appeal would correlate with conscientiousness (*r_p_* = .15, *p* = 4.6^−4^) and disinhibition (*r_p_* = −.11, *p* = 7.1^−3^) received support. However, as was generally the case for PHMs, its appeal also correlated with other traits: positively with adaptive and negatively with maladaptive ones.

The hypotheses that PHM3: Compassionate’s appeal would be predicted by agreeableness (*r_p_* = .37, *p* = 5.6^−18^) and conscientiousness (*r_p_* = .17, *p* = 8.0^−5^) and, negatively, by meanness (*r_p_* = −.31, *p* = 3.3^−13^), Machiavellianism (*r_p_* = −.23, *p* = 1.6^−7^), and SD3 psychopathy (*r_p_* = −.26, *p* = 2.5^−9^) received support; the hypothesis that it would correlate negatively with narcissism (*r_p_* = −.04, *p* = 2.0^−1^) did not. In EAs, PHM3 appeal correlated also with openness (*r_p_* = .15, *p* = 3.3^−4^), disinhibition (*r_p_* = −.24, *p* = 1.7^−8^), and all superfactors.

As predicted, neuroticism correlated with PHM4: avoidant’s appeal (*r_p_* = .13, *p* = 2.2^−3^) but also with all other PHMs, and PHM4 had a stronger correlation with agreeableness (*r_p_* = .24, *p* = 2.7^−8^). Contrary to expectations, boldness did not predict PHM4 ratings (*r_p_* = .01, *p* = 4.6^−1^).

No support emerged for the hypotheses that PHM5: sociable’s appeal would correlate with extroversion (*r_p_* = −.10, *p* = 1.4^−2^), openness (*r_p_* = .06, *p* = 8.2^−2^), and boldness (*r_p_* = .03, *p* = 2.6^−1^). Instead, like the other PHM’s, PHM5 correlated with agreeableness (*r_p_* = .21, *p* = .6.5^−7^) and neuroticism (*r_p_* = .18, *p* = 2.1^−5^) and, negatively, meanness (*r_p_* = −.13, *p* = 2.3^−3^).

### Post Hoc Analyses

Unplanned regression analyses clarified the relative unique contributions of normal-range and dark traits to key health-related variables (Tables S19–S21**)**.

PHM: compassionate was participants’ top choice by far (*n =* 208; more than twice as often as other PHMs). In EAs, participants’ highest ranked PHM predicted no MIP variables. It predicted AMP and SD3 scores except boldness and psychopathy. For example, meanness, *F*(4, 493) = 5.5, *p* = 2.4^−4^, partial η^2^ = .043 (small effect), was lower than average in participants who ranked PHM: compassionate the highest, *t* = −.114, *p* = 8.5^−4^, 95% CI = [−.18, −.05]. Disinhibition, narcissism, and Machiavellianism showed similar patterns.

## Discussion

This research extends personality theory to differences in people’s reactions to a pandemic of communicable SARS. The five-factor, triarchic psychopathy, and Dark Triad models informed such questions as: What traits predict intent for SD and hygiene, endorsement of behavior that risks others’ health, and the appeal of differently phrased PHMs? Such knowledge may aid individual risk prediction (Chapman et al., 2019). It may have applied implications when a minority of people dismisses conventional PHMs (de Bruin et al., 2020).

Twenty-four of the 40 hypotheses received support. This was true particularly for hypotheses using trait theory to predict people’s self-reported current and intended FHB, as well as risks they may create for others. Although most correlations were small (a few were medium), they may reflect meaningful patterns in behavior over time. Self-reported traits predict trait-relevant behavior (Fleeson & Gallagher, 2009), including health behaviors sampled in real time (Kroencke et al., 2019). Furthermore, behavioral intentions likely predict actual behavior (Webb & Sheeran, 2006). The results probably reflect true trait–behavior connections.

### Normal-Range Personality

Regarding the FFM, 8 of the 11 hypotheses received support. In planned and EAs, agreeableness and conscientiousness predicted endorsement of SD and hygiene. This agrees with prior literature on health (Strickhouser et al., 2017), health behaviors (Roberts et al., 2005), and nonrespiratory contagions (Mõttus et al., 2012). Neuroticism may play a small role in hygiene intent, whereas extroversion’s negative links to SD and hygiene vanished after controlling for other traits. Consistent with the EPPM (Witte, 1992), conscientiousness and neuroticism interacted in predicting adaptive responses to the pandemic (perhaps by increasing susceptibility to PHMs). Somewhat consistent with prior research on work and school behavior, conscientiousness and agreeableness showed a trend toward a synergistic interaction.

If such findings replicate, future research should address mechanisms behind them. Such mechanisms may include the empathic concern for others and flexibility in interpersonal behavior entailed in agreeableness (Graziano & Tobin, 2017) and the industrious self-efficacy and self-regulation involved in conscientiousness (Jackson & Roberts, 2017).

### Dark Personality

Regarding triarchic psychopathy and the Dark Triad, 9 of the 12 hypotheses received support. In planned and EAs, meanness and disinhibition (and overall psychopathy) as well as Machiavellianism (to a lesser extent) predicted lower intent for SD and hygiene. Together with boldness, the psychopathy traits predicted endorsement of risky, venturesome behavior (when participants were asked to imagine being disease carriers). This agrees with research on dark personality in relation to health risk (including nonrespiratory communicable disease; Malesza & Kaczmarek, 2019).

As predicted, meanness and disinhibition (and overall psychopathy, but not boldness) predicted endorsement of behavior that puts others at risk of infection (knowingly, and perhaps deliberately). This corroborates the link between psychopathy and unethical everyday behavior (Benotsch et al., 2012; Pletti et al., 2017). In EAs, disinhibition related to all undesirable behaviors, meanness was particularly implicated in putting others at risk, and boldness may be linked to (adaptive) intent for hygiene. This parallels research (in nonforensic samples), whereby boldness either does not predict maladaptive or predicts adaptive outcomes (Berg et al., 2017; Sörman et al., 2016).

Future research may ask whether similar mechanisms explain the links between the two sets of traits (maladaptive vs. adaptive) and health outcomes. Beyond this project’s scope (see Limitations section), the ability of dark traits to predict health risk behavior incrementally (over and above normal traits) merits investigation.

### Appeal of PHMs

Knowledge of the kind presented above becomes useful if it can inform public-health interventions, for example, to limit a pandemic. As noted earlier, researchers are testing personality theory’s ability to inform the tailoring of effective communication in applied contexts. I tested whether personality predicted participant’s ratings of differently phrased PHMs.

This was, indeed, the case, but not always in the predicted manner. Only 8 of the 17 predictions received support. This may reflect methodological limitations. For example, the PHMs may not have been congruent with the self-schemas they were meant to match, or schema-congruence theory cannot encompass self-reported traits and trait-congruent PHMs (York et al., 2012b).

Agreeableness and conscientiousness predicted the appeal of the compassionate PHM (“Help protect the vulnerable!”), conscientiousness of the responsible message (“Take personal responsibility!”), and neuroticism of the avoidant message (“Avoid the disease!”). Psychopathy, meanness, and Machiavellianism negatively predicted the compassionate message’s appeal. However, such findings were message-nonspecific: Overall, adaptive traits predicted PHM’s appeal, and maladaptive traits predicted their nonappeal.

Participants typically chose the compassionate message over others (including the self-centered one: “Keep yourself healthy!”). This parallels finding that appeals to altruism improve hygiene in analog (Betsch et al., 2013) and real-life (Grant & Hofmann, 2011) experiments. Yet, ranking the compassionate message as most effective was linked to lower meanness, disinhibition, narcissism, and Machiavellianism. Thus, appeals to altruism may work for most people but might *backfire* in antagonistic individuals.

### Limitations

The influential personality models that informed this study are not free of potentially controversial assumptions (e.g., Watts et al., 2017). For example, Machiavellianism and psychopathy may not be truly separable (Miller et al., 2016). Indeed, their scores were highly intercorrelated and had similar associations with health-behavior endorsement. The models imply separation between normal-range versus maladaptive traits. However, much of the personality variation they encompass may be due to a smaller set of underlying traits with wide ranges of intensity (Hyatt et al., 2020; Lynam & Miller, 2019).

The nonprobability sample and potentially unusual characteristics of MTurk participants may raise concerns. Reviews of studies on MTurk in personality research (e.g., Chandler & Shapiro, 2016; Miller et al., 2017) have been favorable regarding the measurement, factor structures, and construct validity of normal-range and maladaptive traits (including from five-factor, Dark Triad, and psychopathy-related models).

The sample included participants from across the United States. However, most represented were California (13%) and New York (6%), already heavily affected by the pandemic. In conjunction with the study’s timing, self-selection into research on COVID-19, and the participants’ relatively high education, the sample’s composition may have affected the results. The most likely impact is range restriction of the health and risk behavior scores. Indeed, consistent with polls, most participants highly endorsed health-protective behaviors. Thus, I may have underestimated the magnitude of trait–behavior correlations, perhaps promoting false negative results. This was countered by a respectably large sample. Future research, if using similar methods, may benefit from more sensitive rating scales to mitigate range restriction.

Screening out noncompliant and suspicious responses likely increased measurement reliability, thus promoting power to obtain true positive results. Conversely, screening may have limited the range of irresponsible, disinhibited, antagonistic, and deceptive (i.e., psychopathic) traits, making it harder to detect their links to other variables.

The self-report, cross-sectional, survey method using convenience sampling enabled rapid data collection at a unique historic moment. It also poses limitations, some of which (e.g., measuring intent instead of observing behavior; potential noncompliant responding) have been noted. I used brief personality measures for practicality; however, by limiting content validity and measurement reliability (Crede et al., 2012), this may have caused inaccurate estimation (most likely underestimation) of some correlations. Also by necessity, the health-behavior and PHM-appeal measures had not been pilot tested or previously validated. Besides age, gender, and health status, the study did not include potentially relevant predictors or suppressors, such as COVID-19 knowledge accuracy, misinformation susceptibility, political affiliation (Allyn & Sprunt, 2020), and regional policies. Furthermore, the measures were acontextual vis-à-vis interpersonal factors like kinship, in-group/out-group membership, and situational factors that may interact with personality.

## Conclusion

In summary, early in the pandemic, prosocial traits likely facilitated SD and hygiene, and antagonistic traits likely detracted from adaptive behavior and promoted harm to people’s health. Distinctly antagonistic persons may have disregarded or acted contrary to public-health appeals for altruistic behavior.

This is an early demonstration that personality may matter in understanding communicable respiratory disease. More generally, personality has implications for contagious diseases and public-health communication. The findings, if replicated, may find applications in public-health messaging, individual risk prediction, and doctor–patient communication.

The results *do not* mean that it is mostly irresponsible and inconsiderate people who spread viruses. The correlations were often small, and the traits’ scientific conceptualizations are not quotidian judgments about character. The results *do not* mean that people who contract a disease like COVID-19 have maladaptive traits. The findings do invite further research on personality in public health.

## Supplementary Material

Supplementary material

Supplementary material

Supplementary material
