# Supplementary material for: Adaptive and Dark Personality in the COVID-19 Pandemic: Predicting Health-Behavior Endorsement and the Appeal of Public-Health Messages
Source: Soc Psychol Personal Sci. 2021 Jul;12(5):697–707. doi: 10.1177/1948550620936439 (PMC7342937; doi:10.1177/1948550620936439)
Supplement: Supplementary material [file registration-screenshots_(1).pdf]

OSF REGISTRIES Help Donate

Personality and responses to the coronavirus pandemic

Public registration

Overview

Files

Wiki

Components

Links

Analytics

Comments

Summary

Provide a narrative summary of what is contained in this registration or how it differs from prior registrations. If this project contains documents for a preregistration, please note that here.

General Background

The literature on general personality and physical health has focused primarily on broad health outcomes and, to an extent, on specific health behaviors. Responses to a pandemic, particularly with regard to hygiene and social distancing, have not been examined. The literature on psychopathy and the dark triad has linked these characteristics to risk-taking and, in few studies, to physical health outcomes, and there is an increasing interested in psychopathy, the dark triad, and everyday behavior. These traits have not yet been linked to hygiene and social distancing in a pandemic. Finally, personality has been linked to responses to political and commercial messaging, but not to public health messaging. The emergent coronavirus pandemic presents an opportunity to test hypotheses in this area at a time when most people in the U.S. are at least somewhat informed about the pandemic and are still adapting to new demands for social distancing, increased hygiene, and public-health messages.

The study being registered will link general personality (the Big Five), the triarchic psychopathy model, and the dark triad to self-reported current and intended hygiene and social distancing, to self-reported endorsement of intent to harm others by spreading the infection, and to the personal appeal of public-health messaging about how to protect oneself and others from the virus. Specific predictions are listed following the method description.

Method Basics

Research participants will be up to 500 Mechanical Turk Workers, to be compensated $1.5 for a 15-min. survey. The survey will consist of ad hoc questionnaires about hygiene, social distancing, social and self-protective behaviors, intent to harm others, the mini-IPIP scales (Donnellan et al., 2006), Abbreviated Psychopathy Measure (Semel, 2018), Short Dark Triad (Jones & Paulhus, 2014), and brief scales to measure the persuasiveness, likelihood to take seriously, and likelihood to impact behavior of each of five public-health messages. The messages have been written ad hoc and based to theory to use language that would especially appeal to or be ego-syntonic with

Contributors

[redacted]

Description

Adaptive and Dark Personality in the Covid-19 Pandemic: Predicting Health-behavior Endorsement and the Appeal of Public-health Messages

Registration type

Open-Ended Registration

Date registered

March 19, 2020

Date created

March 19, 2020

Registered from

[redacted]

Category

Project

Registration DOI

No DOI assigned

Publication DOI

No publication DOI

Subjects

Psychology

Social and Behavioral Sciences

theory to use language that would especially appeal to or be ego-synt onic with narcissistic/Machiavellian, conscientious/ overcontrolled, agreeable/compassionate, avoidant/contamination-averse. and extraverted/sociableperson alities. Composite scores will be created from the personality questionnairesaccor ding to their conventional scoring guidelines. Composite scores will be created for the health-related questionnairesto create the followingvariables: Current social distancing; current hygiene: intent to socially distance:intent for hygiene: willingnessto harm others; message app eal (separately for each of the five messages). The correlational analyses linking personality variables to the health-related composites will control for age. sex, and the extent to which participants report having chronic health conditions that may make them especially susceptibleto coronavirus (on a scale from 0-2).

Power and Data Cleaning

Partial correlations will be considered significant at p <= .01 (one-tailed). This corresponds to .85 power at alpha = .01 (one-tailed) for an r = .16 with N = 430. Wewill attempt to obtain valid data from upto 500participants based on budget limitations. Realistically, we expect that approximately 60 out of 500 participants will provide data with questionable validity.This will bedetermined through theuse of validity and consistency check.and survey-completion times. We will plot the survey-completion times and look for an inversion point th at distinguishes extremely fast completion times frommore realistic completiontimes. We anticipate excluding the participants with the 25 lowest completion times, but we must inspectth e actual completion-time data before committing to this decision. Wewill examine responses to three pairs of inconsistency check questions. to three validity questions (e.g.. I am answering these questions tru thfully), and to three questions expected to produce nearly constant responses.Wewill examine the distribution of theseinconsistency/validity data and exclude extremely inconsistenUinvalid responses. We anticipate excluding participants (probably around 50-60) who fail 2-3 of th e inconsistency checkand 2-3 of the validity checks, but we must inspect the actual inconsistency/validity data before committing to t his decision. Wedo not anticipate engaging in other data cleaning or in the identification or selection of outliers.

Specific Predictions

Sel f-reported inclinatlon to engagein social distancing will correlate with:

- Conscientiousn ess. Agreeableness, Neuroticism. Boldn ess (negatively), Disinhibi tion (negat ively), and Psychopathy (negatively)

Sel f-reported inclination to engage in hygiene will correlate with:

- Conscientiousn ess. Agreeableness, Disinhibit ion (negatively). Meanness(negatively)

Self-reported actual engagement in social distancing will correlate with:

- Conscientiou sness. Agreeableness. Extraver sion (negatively). Neuroticlsm. Boldness (negatively), Disinhibition (negatively)

Sel f-reported actual engagement in hygiene practice will correlate with:

- Conscientiou sness. Agreeableness, Narcissism (because of a desirability bias) and Boldness(for thesame reason)

Sel f-reported willingness to deliberately infect others will correlate with:

- Meanness, Disinhibition. Machiavellianism. Psychopathy

Finally. appeal of the five public-health messages will corr elate differe ntially wit h the traits:

The narcissistic/Machiavellian message: Narcissism. Meanness, Machiavellianism.

Health Psychology

Personality and Social Contexts

Affiliated institutions

This registration has no affiliated institut ions

Lic e nse

CC-By Attribution 4.0 International

Tags

abbreviated measure of psychopathy

coronavirus Covid-19

dark triad disinhibition

endangering othe rs

extended parallel processmodel

five-factor model

health behavior

health-risk behavior

Infectious respiratory disease

Machiavellianism meanness

pandemic psychopathy

public-health messaging

respiratory disease

schema-congruency theory

shortdark triad

b·iarchic model of psychopathy

Cita tio n

[redacted]

Psychopathy;

The conscientious/overcontrolled message: Conscientio usness and Disinhibition {negat ive);

The agreeab le/compass ionate message: Conscientiousn ess, Agreeableness, Narcissism {negative), Machiavellianism {negative), Psychopathy (negative), and Meanness {negative);

The avoidanUfearful message: Neuroticism and Boldness {negative)

The extraverted message Extraversion, Boldness, and Openness.

How does this summary differ from prior registrations?

There are no prior registrations of similar projects. This is a unique project that takes advantage of the emergent coronaviruspandemic. The IRSproposal wassubmitted on March 19, 2020, and data collection is expected to take placebefore the end of March, 2020 {pending IRS and budgetapproval).

Add supplemental files or additional information

No filesselected

Copyright © 2011-2020Center for Open Science | Terms of Use | Privacy Poli cy | Status | API
TOP Guid elines | Repro du cibility Project: Psychology | Repro d u cibility Project: Cancer Biology
