# Supplementary material for: Adaptive and Dark Personality in the COVID-19 Pandemic: Predicting Health-Behavior Endorsement and the Appeal of Public-Health Messages
Source: Soc Psychol Personal Sci. 2021 Jul;12(5):697–707. doi: 10.1177/1948550620936439 (PMC7342937; doi:10.1177/1948550620936439)
Supplement: Supplementary material [file supplementary-source-masked_(1).pdf]

Supplementary Materials

for

Adaptive and Dark Personality in the Covid-19 Pandemic:

Predicting Health-behavior Endorsement and the Appeal of Public-health Messages

2020

Table of Contents

Table S1: Distribution of Effort Check Data....................3

Table S2: Distribution of Invalid Responding Data....................4

Table S3: Distribution of the Inconsistency Check Data....................5

Table S4: Distribution of Completion Times Below the Median....................6

Table S5: Principal Components Analysis of Current Health Behavior (CHB)....................7

Table S6: Principal Components Analysis of Future Health Behavior (FHB)....................8

Table S7: Principal Components Analysis of Carrier Pretend Scenario Ratings (CPS)....................9

Table S8: Participant Characteristics....................10

Table S9: Self-reported Location by U.S. State....................11

Table S10: Descriptives for Health Behavior Endorsement and Message Appeal Variables 12

Table S11: Descriptives for Personality Variables....................13

Figure S1: The Compassionate and Sociable Messages Were the Most and Least Favored 14

Table S12: Bivariate Correlations among Health Behavior Endorsement and Public-health Message Appeal Variables....................15

Table S13: Bivariate Correlations among Personality Variables....................16

Table S14: Bivariate Correlations among Main Study Variables and Covariates....................17

Table S15: Descriptive Statistics for Personality Superfactors....................18

Table S16: Exploratory Analyses with Personality Superfactors and Health Behavior....................19

Table S17: Exploratory Analyses with Personality Superfactors and Message Appeal....................20

Table S18: Exploratory Multiple Regression Analyses Predicting Message Appeal....................21

Figure S2: Conscientiousness and Neuroticism Interact in Predicting Current Social Distancing 22

Figure S3: Conscientiousness and Agreeableness as Related to Future Social Distancing....................23

Table S19: Unplanned Multiple Regression Analyses Predicting Key Health-related Variables from Normal-range Personality Dimensions....................24

Table S20: Unplanned Multiple Regression Analyses Predicting Health-related and Harmful Behavior Endorsement from the Triarchic Model of Psychopathy Dimensions....................25

Table S21: Unplanned Multiple Regression Analyses Predicting Health- related and Harmful Behavior Endorsement from the Dark Triad of Personality Dimensions....................26

Appendix SA: Text of the Public-health Messages 27

Table S1

Distribution of Effort Check Data (N = *652*)

**Bootstrap (1000)**		**Bias**	***SE***	**95% C.I.**	
M	0.055	0.000	0.009	0.038	0.075
Md	0	0	0	0	0
SD	0.229	-0.001	0.018	0.192	0.264
**Skewness**	3.904	0.054	0.402	3.230	4.819
***SE* Skewness**	0.096				
**Kurtosis**	13.280	0.589	3.263	8.461	21.292
***SE* Kurtosis**	0.191				
Min.	0				
Max.	1				

**Frequencies**	***n***	**%**
0	616	94.5
1	36	5.5
N	652	100

Note: 1 = failure of any of four effort checks. Effort checks were simple analogies, like: "*Sheep* is to *lamb* as ___ is to a *calf.*"

Table S2

Distribution of Invalid Responding Data (N = *616)*

**Bootstrap (1000)**		**Bias**	***SE***	**95% C.I.**	
M	0.395	0.001	0.032	0.335	0.466
Md	0	0	0	0	0
SD	0.837	0.000	0.051	0.746	0.939
**Skewness**	2.637	-0.014	0.179	2.293	2.994
***SE* Skewness**	0.098				
**Kurtosis**	7.543	-0.096	1.294	5.159	10.358
***SE* Kurtosis**	0.197				
***PR* = 95**	2	0.147	0.345	2.000	3.000
***PR* = 98**	3	0.275	0.424	3.000	4.000
***PR* = 99**	4	-0.057	0.267	3.000	4.000
Min.	0				
Max.	5				
**Possible range**	0-6				

PR = Percentile rank. Validity checks were responses with scores beyond the midpoint in the undesirable direction on such question as “I am answering these questions thoughtfully.”

Table S3

Distribution of the Inconsistency Check Data (N = *562*)

**Bootstrap (1000)**		**Bias**	***SE***	**95% C.I.**	
M	1.867	-0.001	0.051	1.767	1.970
Md	2.000	-0.022	0.143	2.000	2.000
Mode	1.000				
SD	1.226	-0.005	0.060	1.105	1.340
**Skewness**	1.509	-0.016	0.131	1.220	1.731
***SE* Skewness**	0.103				
**Kurtosis**	3.224	-0.057	0.553	2.131	4.284
***SE* Kurtosis**	0.206				
Min.	0				
Max	7				
***PR* = 95**	4	0.100	0.293	4.000	5.000
***PR* = 98**	6	-0.282	0.445	5.000	6.000
***PR* = 99**	6.370	0.066	0.465	6.000	7.000

PR = Percentile rank.

**Inconsistency**	**n**	**%**	**Cum. %**
0	28	5	5
1	228	40.6	45.6
2	186	33.1	78.6
3	71	12.6	91.3
4	27	4.8	96.1
5	9	1.6	97.7
6	8	1.4	99.1
7	5	0.9	100
**N**	562	100	

Note: Inconsistency scores are based on the sum of the absolute values of the differences between the items from three pairs of nearly identical items after any necessary reverse-coding.

Table S4

Distribution of Completion Time Below the Median (N = 540)

```
Completion time (min.) Stem-and-Leaf Plot

 Frequency    Stem &  Leaf

     1.00 Extremes    (=<2.5)
     1.00        5 .  4
     3.00        5 .  779
     4.00        6 .  2344
     6.00        6 .  678999
    11.00        7 .  12222334444
    11.00        7 .  56667788889
    13.00        8 .  0000222222334
    16.00        8 .  5666677888888999
    20.00        9 .  01111111122222233444
    21.00        9 .  555666667777888999999
    26.00       10 .  00000001111222222233333344
    29.00       10 .  55555555566667777788888899999
    15.00       11 .  011112223333344
    17.00       11 .  55566777778889999
    18.00       12 .  000011111222333334
    20.00       12 .  56666777777777789999
    22.00       13 .  0000001111122222444444
    16.00       13 .  5566666777788889

 Stem width:      1.00
 Each leaf:       1 case(s)
```

Table S5

*Principal Components Analysis with Varimax Rotation of Current Health Behavior (*N *= 502)*

Abbreviated item	Component		Variance Explained			Commu-
	1	2	#	Eigenvalue	%	nality
1. Limit contact	.83		1	5.26	52.57	.75
3. Stay at home	.83		2	1.22	12.16	.71
4. Avoid social activities	.77		3	0.73	7.30	.62
5. Avoid visiting relatives	.74		4	0.57	5.72	.66
2. Keep a 6-foot distance	.71	.36	5	0.51	5.08	.64
6. Protect older people	.70	.33	6	0.42	4.23	.60
7. Wash hands frequently		.81	7	0.39	3.88	.72
9. Disinfect surfaces		.81	8	0.33	3.29	.69
8. Avoid touching face		.80	9	0.31	3.08	.71
10. Cover one's cough	.30	.54	10	0.27	2.69	.39

Note: Loadings > .3 were omitted.

Table S6

Principal Components Analysis of Future Health Behavior (N = *502*)

Abbreviated item	Component			Variance Explained			Commu-
	1	2	3	#	Eigenvalue	%	nality
1. Limit contact	.83	-.30		1	8.54	60.97	.87
3. Stay at home	.82	-.33		2	1.82	13.00	.83
4. Avoid visiting relatives	.78			3	.97	6.91	.72
2. Keep a 6-foot distance	.75		.43	4	.47	3.37	.81
6. Protect older people	.75		.42	5	.39	2.81	.80
5. Avoid social activities	.74	-.45		6	.36	2.55	.81
13. Go on a new date		.90		7	.32	2.29	.91
14. Sex with someone new		.89		8	.30	2.17	.88
11. Go to large social event		.88		9	.23	1.66	.85
12 .Go to small social event	-.36	.84		10	.18	1.29	.85
9. Disinfect surfaces			.82	11	.17	1.18	.76
8. Avoid touching face			.82	12	.14	1.00	.80
7. Wash hands frequently	.34		.79	13	.08	0.57	.80
10. Cover one’s cough	.42	-.40	.56	14	.03	0.24	.80

Note: Loadings > .3 were omitted.

Table S7

Principal Components Analysis of Carrier Pretend Scenario Ratings (N = *502*)

	Item	Component		Variance Explained			Commu-
		1	2	#	Eigenvalue	%	nality
18.	Purposefully touch, spit on, or sneeze on high-use surfaces in public	.91		1	5.91	32.85	.88
17.	Purposefully touch, spit on, or sneeze on high-use surfaces at work	.91		2	2.40	13.31	.88
15.	Purposefully shake hands with, hug, or kiss someone you hate	.73		3	1.61	8.93	.80
7.	Go to a social event or a party	.73		4	1.15	6.38	.69
12.	If you get the chance, go on an in-person date with someone new	.70	(.39)	5	1.03	5.72	.77
8.	Shake hands with strangers	.62	(.50)	6	0.94	5.22	.70
16.	Sneeze or spit on the personal belongings of some you hate	.61		7	0.91	5.06	.87
9.	Shake hands with people you know well		.75	8	0.78	4.32	.81
10.	Shake hands with people you care about	(.43)	.68	9	0.67	3.71	.80
13.	If you get the chance, go to an in-person job interview		.67	10	0.53	2.93	.50
11.	Hug and/or kiss people that you are close with		.64	11	0.49	2.74	.62
4.	Wear a mask outside your home (assuming you have masks) (R)		.54	12	0.43	2.37	.67
5.	Go to work (assuming you work and not from home)		.51	13	0.39	2.17	.49
6.	Go out in public to take care of business that isn't urgent		.48	14	0.29	1.63	.56
3.	Wear a mask at home (assuming you have masks) (R)		.48	15	0.23	1.26	.59
14.	If you get the chance, have sex (in person, not online) with someone new	.42	(.47)	16	0.15	0.85	.64
1.	Go to seek medical attention, but first call ahead (R)			17	0.10	0.57	.48
2.	Go to seek medical attention, without calling ahead			18	0.00	0.00	.33

Note: Item 10 was allocated to the first component in scale creation for conceptual coherence.

Table S8

*Participant Characteristics (*N *= 502)*

**Sex**	51% male	**Socioeconomic status (current)**	
	49% female	Poor	8.2%
**Age**	*M* = 41, *SD* = 11, *Md* = 40	Working class	33.9%
	Range = 21 – 76 years	Lower middle class	15.9%
**Education**	*M* = 15, *SD* = 2.04, *Md* = 16.0	Middle class	35.3%
**Race/Ethnicity**		Upper middle class	6.8%
77.5%	White/Caucasian	Higher class	0.0%
10.0%	Asian/Asian-American	**Employment status**	
6.2%	Black/African-American	Full-time employed	60.4%
3.8%	Latinx/Hispanic	Part-time employed	8.8%
0.6%	Native American	Self-employed	21.3%
2.0%	Other	Full-time student	0.6%
**Relationship status**		Unemployed	2.8%
47.2%	single	Disabled	1.2%
36.3%	married	Retired/Retired but working	3.6%
9.4%	divorced	Other	1.7%
5.2%	life-partnered	**Covid-10 effect on work**	
0.6%	separated	No effect	57.0%
1.6%	widowed	More hours/extra shifts	4.6%
**Work from home?**		Fewer hours/fewer shifts	22.5%
33.3%	Yes, normally from home	Furlough	5.8%
25.7%	Yes, because of coronavirus	Laid off	3.0%
40.2%	No / Not applicable	Other	6.8%
**Covid-19 diagnosis history**		**Covid-19 risk: chronic condition**	
1	"Yes"	No	72.5%
20	"No, but WE think WE had it"	Not sure	6.8%
481	"No"	Yes	20.7%

Table S9

Self-reported Location by U.S. State

State	n	%	State (cont.	n	%
Alabama	6	1.2	Nevada	7	1.4
Arkansas	0	0	New Hampshire	1	0.2
Arizona	13	2.6	New Jersey	15	3
California	63	12.5	New Mexico	3	0.6
Colorado	12	2.4	New York	29	5.8
Connecticut	7	1.4	North Carolina	25	5
Delaware	1	0.2	North Dakota	0	0
District of Columbia	1	0.2	Ohio	14	2.8
Florida	35	7	Oklahoma	7	1.4
Georgia	14	2.8	Oregon	4	0.8
Hawaii	4	0.8	Pennsylvania	31	6.2
Idaho	0	0	Puerto Rico	0	0
Illinois	15	3	Rhode Island	3	0.6
Indiana	2	0.4	South Carolina	8	1.6
Iowa	2	0.4	South Dakota	0	0
Kansas	3	0.6	Tennessee	8	1.6
Kentucky	12	2.4	Texas	27	5.4
Louisiana	7	1.4	Utah	3	0.6
Maine	2	0.4	Vermont	3	0.6
Maryland	7	1.4	Virginia	13	2.6
Massachusetts	10	2	Washington	13	2.6
Michigan	23	4.6	West Virginia	2	0.4
Minnesota	10	2	Wisconsin	20	4
Mississippi	0	0	Total	501	99.8
Missouri	8	1.6	Missing	1	0.2
Montana	4	0.8	Sample	502	100
Nebraska	4	0.8			

Table S10

Descriptives for Health Behavior Endorsement and Message Appeal Variables (N = *502*)

	Items	Scale	*M*	95% C.I.[2]	*Md*	*SD*	α
**Health Behavior Endorsement**				bias-corrected			
Current Social Distancing	6	0-3	2.55	[2.5 - 2.6]	2.7	0.57	.89
Current Hygiene	4	0-3	2.31	[2.3 - 2.4]	2.5	0.63	.80
Future Social Distancing	6	0-4	3.55	[3.5 - 3.6]	3.8	0.83	.95
Future Hygiene	4	0-4	3.31	[3.2 - 3.4]	3.8	0.89	.87
Future Venturous Behavior	4	0-4	0.24	[0.2 - 0.3]	0.0	0.79	.95
Carrier Scenario: Protecting Others	8	0-3	0.03	[0.0 - 0.1]	0.0	0.14	.65
Carrier Scenario: Harmful Behavior	8	0-3	2.65	[2.6 - 2.7]	2.8	0.36	.74
**Public-health Message (PHM) Appeal[1]**							
Message 1: Self-centered	3	0-100	77.11	[75 - 79]	83.3	22.44	.94
Message 2: Responsible	3	0-100	78.41	[77 - 80]	83.3	22.41	.94
Message 3: Compassionate	3	0-100	83.73	[82 - 86]	90.0	19.43	.93
Message 4: Avoidant	3	0-100	76.61	[75 - 79]	83.3	23.31	.95
Message 5: Sociable	3	0-100	66.29	[64 - 69]	73.3	28.74	.94
**PHM Rankings**							
Message 1: Self-centered – rank	1	1-5	2.71	[2.6 - 2.8]	3	1.16	-
Message 2: Responsible – rank	1	1-5	2.75	[2.7 - 2.9]	3	1.22	-
Message 3: Compassionate – rank	1	1-5	2.22	[2.1 - 2.3]	2	1.28	-
Message 4: Avoidant – rank	1	1-5	3.20	[3.1 - 3.3]	3	1.37	-
Message 5: Sociable - rank	1	1-5	4.13	[4.0 - 4.2]	5	1.28	-

[1] Messages were presented for evaluation in a random order.

[2] Bootstrapping estimates based on 1000 iterations.

Table S11

*Descriptives for Personality Variables (*N *=502*)

	Items	Scale	*M*	95% C.I.[**]	*Md*	*SD*	α
**Mini-IPIP[*]**				bias-corrected			
Agreeableness	4	0-4	2.92	[2.8 – 3.0]	3.0	0.90	.88
Conscientiousness	4	0-4	2.93	[2.9 – 3.0]	3.0	0.80	.77
Extraversion	4	0-4	1.38	[1.3 - 1.5]	1.0	1.10	.89
Neuroticism	4	0-4	1.40	[1.3 - 1.5]	1.3	1.01	.84
Openness	4	0-4	3.00	[2.9 - 3.1]	3.0	0.86	.80
**AMP[*]**							
Boldness[1]	11	0-3	0.97	[0.9 – 1.0]	0.91	0.56	.86
Meanness[1]	11	0-3	0.44	[0.4 - 0.5]	0.27	0.47	.88
Disinhibition[1]	11	0-3	0.50	[0.5 - 0.5]	0.36	0.45	.87
**SD3[*]**							
Narcissism	9	0-4	1.39	[1.3 - 1.5]	1.33	0.77	.84
Machiavellianism	9	0-4	1.75	[1.7 - 1.8]	1.67	0.74	.84
Psychopathy	9	0-4	0.87	[0.8 - 0.9]	0.78	0.62	.79

[*]**Mini-IPIP** = Mini-Internal Personality Item Pool (Donnellan et al., 2006); **AMP** = Abbreviated Measure of Psychopathy (Semel, 2018); **SD3** = Short Dark Triad (Jones & Paulhus, 2014).
[1]Scores reversed for interpretability.
[**] Bootstrapping estimates based on 1000 iterations.

Figure S1

The Compassionate and Sociable Messages Were the Most and Least Favored

1 = Self-centered; 2 = Responsible; 3 = Compassionate; 4 = Avoidant; 5 = Sociable.

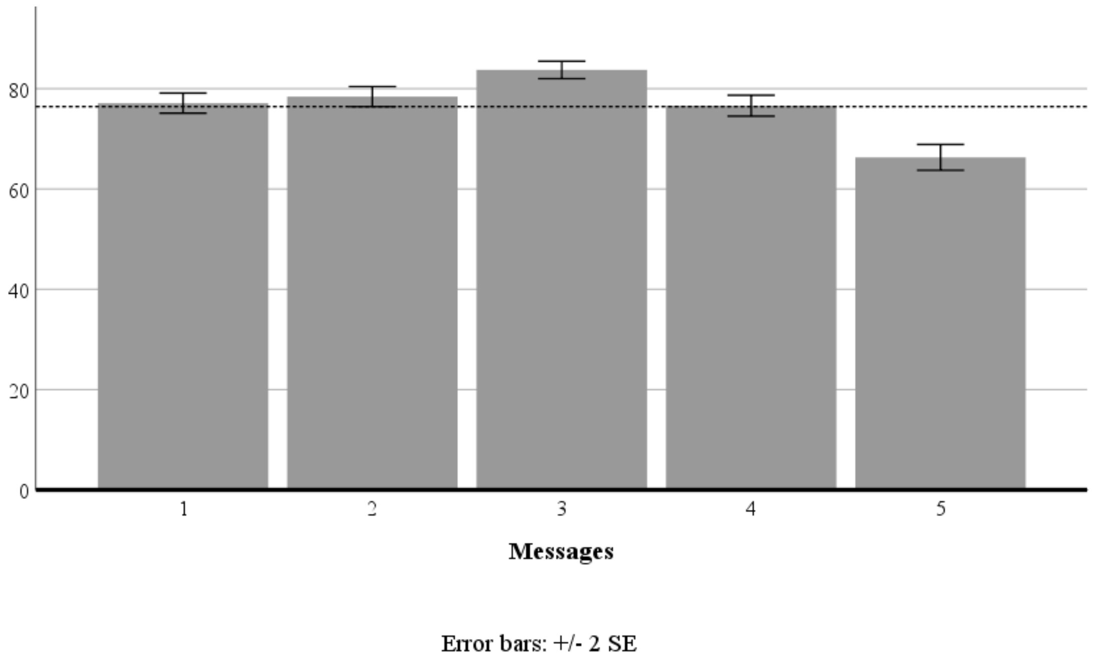

Table S12

*Bivariate Correlations among Health Behavior Endorsement and Public-health Message Appeal Variables, Two-tailed (*N *= 502)*

**Pearson correlations**		**1**	**2**	**3**	**4**	**5**	**6**	**7**	**8**	**9**	**10**	**11**
**1. CHB: SD**	***r***	-										
	p	-										
**2. CHB: H**	***r***	.61										
	p	1.3E-51										
**3. FHB: SD**	***r***	.58	.38									
	p	1.7E-46	5.2E-19									
**4. FHB: H**	***r***	.41	.69	.73								
	p	7.1E-22	3.5E-73	2.2E-84								
**5. FHB: VB**	***r***	-.12	-.08	-.62	-.50							
	p	6.0E-03	6.4E-02	8.3E-54	3.1E-33							
**6. CS: HB**	***r***	-.25	-.17	-.19	-.17	.20						
	p	1.6E-08	9.4E-05	1.4E-05	2.0E-04	8.3E-06						
**7. CS: PO**	***r***	.30	.37	.23	.29	-.13	-.47					
	p	4.7E-12	1.7E-17	2.8E-07	1.7E-11	4.0E-03	1.4E-28					
**8. M1: SC**	***r***	.42	.46	.29	.38	-.05	-.14	.23				
	p	1.2E-22	9.3E-28	1.6E-11	1.8E-18	3.1E-01	2.3E-03	1.1E-07				
**9. M2: R**	***r***	.50	.51	.36	.42	-.09	-.16	.24	.75			
	p	1.7E-33	3.1E-34	1.0E-16	1.6E-22	5.0E-02	2.5E-04	3.1E-08	2.0E-93			
**10. M3: C**	***r***	.54	.52	.36	.42	-.08	-.15	.23	.66	.69		
	p	8.5E-39	8.0E-36	3.0E-17	5.7E-23	8.8E-02	8.0E-04	2.4E-07	4.5E-65	2.2E-72		
**11. M4: A**	***r***	.49	.45	.33	.37	-.05	-.13	.20	.78	.75	.67	
	p	5.0E-31	1.6E-26	5.0E-14	1.1E-17	2.3E-01	2.5E-03	5.8E-06	5.5E-105	3.8E-90	1.7E-65	
**12. M5: SO**	***r***	.37	.38	.21	.27	.03	-.11	.17	.59	.60	.54	.60
	p	5.5E-18	3.1E-18	1.5E-06	1.6E-09	4.7E-01	1.3E-02	1.3E-04	4.5E-48	1.2E-50	1.4E-38	1.3E-50

Note: CHB = Current Health Behavior (SD = Social Distancing, H = Hygiene); **FHB** = Future Health Behavior (VB = Venturous Behavior); CS = Carrier Scenario (HB = Harmful Behavior, PO = Protecting Others); **M** = Public-health Message (SC = Self-centered, R = Responsible, C = Compassionate, A = Avoidant, SO = Sociable). **Note:** $p < .01$ = 1.0E-2, $p < .001$ = 1.0E-3.

Table S13

Bivariate Correlations among Personality Variables, Two-tailed (N = *502)*

		1: MIP A	2	3	4	5	6	7	8	9	0	11	12	13	14	15
**2. MIP C**	***r***	.18														
	p	6.0E-05														
**3. MIP E**	***r***	.30	.18													
	p	1.0E-11	7.7E-05													
**4. MIP NE**	***r***	-.13	-.28	-.38												
	p	4.2E-03	2.6E-10	2.4E-18												
**5. MIP O**	***r***	.35	.05	.30	-.12											
	p	5.0E-16	2.6E-01	3.5E-12	5.6E-03											
**6. MIP ST**	***r***	.64	.67	.43	-.73	.26										
	p	3.2E-58	9.9E-67	1.0E-23	1.1E-84	2.8E-09										
**7. MIP PL**	***r***	.40	.15	.86	-.33	.75	.44									
	p	2.5E-20	8.4E-04	4.3E-145	5.7E-14	5.3E-93	7.7E-25									
**8. MIP PF**	***r***	.62	.51	.74	-.64	.58	.87	.82								
	p	2.2E-54	6.3E-34	4.3E-87	2.3E-59	1.2E-45	4.7E-157	3.5E-124								
**9. AMP B**	***r***	-.10	.00	.50	-.27	.26	.10	.49	.33							
	p	2.6E-02	9.6E-01	1.3E-33	1.1E-09	1.8E-09	2.6E-02	7.5E-32	3.0E-14							
**10. AMP M**	***r***	-.60	-.21	-.07	.24	-.18	-.51	-.15	-.40	.37						
	p	4.2E-50	1.7E-06	1.0E-01	8.4E-08	7.7E-05	4.0E-35	1.0E-03	3.1E-21	5.3E-18						
**11. AMP D**	***r***	-.27	-.41	.11	.21	.01	-.43	.08	-.23	.53	.65					
	p	7.5E-10	5.9E-22	1.5E-02	1.4E-06	7.7E-01	1.2E-23	6.6E-02	3.2E-07	3.4E-38	5.0E-62					
**12. AMP Tot.**	***r***	-.38	-.23	.24	.05	.06	-.31	.20	-.09	.80	.80	.87				
	p	2.1E-18	1.3E-07	3.1E-08	2.9E-01	1.9E-01	1.1E-12	6.0E-06	5.0E-02	3.3E-114	8.6E-113	9.2E-154				
**13. SD3 Nar**	***r***	.03	.12	.60	-.19	.23	.17	.54	.40	.69	.28	.33	.55			

	p	5.6E-01	8.1E-03	2.5E-51	1.7E-05	1.7E-07	1.5E-04	8.3E-40	6.1E-21	4.1E-72	3.5E-10	6.3E-14	3.2E-40			
**14. SD3 Mch**	***r***	-.37	-.16	-.04	.22	-.10	-.37	-.08	-.28	.34	.63	.46	.57	.37		
	p	1.0E-17	4.6E-04	3.2E-01	5.4E-07	3.3E-02	1.1E-17	6.4E-02	1.9E-10	6.0E-15	4.7E-56	1.0E-27	2.4E-44	4.5E-18		
**15. SD3 P**	***r***	-.35	-.28	.14	.19	.01	-.39	.10	-.19	.53	.67	.72	.77	.42	.62	
	p	7.6E-16	1.7E-10	2.3E-03	2.8E-05	7.8E-01	5.1E-20	2.4E-02	1.2E-05	5.4E-38	1.8E-67	4.5E-81	3.6E-100	1.7E-22	9.5E-56	
**16. SD3 DF**	***r***	-.28	-.12	.30	.08	.07	-.23	.24	-.01	.65	.64	.61	.77	.76	.83	.82
	p	3.3E-10	8.9E-03	5.6E-12	8.0E-02	1.4E-01	2.4E-07	3.0E-08	8.0E-01	1.5E-61	3.4E-59	4.8E-52	2.1E-100	2.2E-96	5.7E-128	2.5E-124

Note: **MIP** = Mini-IPIP (A = Agreeableness, C = Conscientiousness, E = Extraversion, NE = Neuroticism, O = Openness, ST = Stability, PL = Plasticity, PF = General P Factor); **AMP** = Abbreviated Measure of Psychopathy (B = Boldness, M = Meanness, D = Disinhibition); **SD3** = Short Dark Triad (Nar = Narcissism, Mch = Machiavellianism, P = Psychopathy, DF = Dark Factor. **Note:** $p < .01$ = 1.0E-2, $p < .001$ = 1.0E-3.

Table S14

*Bivariate (Pearson) Correlations among Main Study Variables and Key Covariates, Two Tailed (*N *= 502)*

		Gender[1]	Age	Risk[2]			Gender	Age	Risk			Gender	Age	Risk
**CHB: SD**	***r***	.19	.11	.17	**MIP: A**	***r***	.26	.09	.05	**AMP: B**	***r***	-.32	-.18	-.08
	p	1.2E-05	1.1E-02	1.5E-04		***p***	4.5E-09	5.4E-02	2.8E-01		***p***	2.5E-13	3.5E-05	7.9E-02
**CHB: H**	***r***	.19	.04	.11	**MIP: C**	***r***	.04	-.04	-.08	**AMP: M**	***r***	-.23	-.22	-.03
	p	2.5E-05	3.3E-01	1.4E-02		***p***	3.6E-01	3.2E-01	8.8E-02		***p***	2.7E-07	8.7E-07	5.2E-01
**FHB: SD**	***r***	.20	.12	.11	**MIP: E**	***r***	-.04	.00	-.09	**AMP: D**	***r***	-.21	-.19	-.04
	p	1.0E-05	5.3E-03	1.6E-02		***p***	4.1E-01	9.7E-01	5.3E-02		***p***	1.8E-06	1.9E-05	3.6E-01
**FHB: H**	***r***	.22	.06	.09	**MIP: NE**	***r***	.16	-.08	.13	**AMP: Tot.**	***r***	-.31	-.24	-.06
	p	8.4E-07	1.6E-01	5.7E-02		***p***	4.5E-04	8.1E-02	3.5E-03		***p***	7.1E-13	6.4E-08	1.6E-01
**FHB: VB**	***r***	-.08	-.05	-.05	**MIP: O**	***r***	-.06	-.01	.05	**SD3: Nar**	***r***	-.19	-.13	-.12
	p	9.0E-02	2.6E-01	3.0E-01		***p***	2.1E-01	8.1E-01	2.7E-01		***p***	1.7E-05	3.5E-03	6.4E-03
**CS: HB**	***r***	-.11	-.16	-.04	**MIP: ST**	***r***	.06	.07	-.08	**SD3: Mch**	***r***	-.28	-.23	-.04
	p	1.2E-02	2.9E-04	3.6E-01		***p***	1.9E-01	1.4E-01	7.1E-02		***p***	2.9E-10	2.5E-07	3.6E-01
**CS: PO**	***r***	.12	.02	.03	**MIP: PL**	***r***	-.06	.00	-.03	**SD3: P**	***r***	-.41	-.24	-.04
	p	9.3E-03	6.2E-01	4.8E-01		***p***	2.1E-01	9.1E-01	4.6E-01		***p***	9.4E-22	3.5E-08	4.2E-01
**M1: SC**	***r***	.14	.24	.10	**MIP: PF**	***r***	.01	.04	-.07	**SD3: DF**	***r***	-.35	-.25	-.09
	p	2.0E-03	5.0E-08	2.0E-02		***p***	8.9E-01	3.8E-01	1.2E-01		***p***	2.5E-16	2.6E-08	5.6E-02
**M2: R**	***r***	.14	.25	.15	**M4: Av**	***r***	.14	.23	.16					
	p	1.2E-03	2.1E-08	7.9E-04		***p***	1.2E-03	1.4E-07	4.1E-04					
**M3: Com**	***r***	.21	.22	.13	**M5: S**	***r***	.10	.15	.15					
	p	2.2E-06	3.9E-07	2.9E-03		***p***	2.9E-02	7.7E-04	7.8E-04					

Note: [1]**Gender** = identified gender (0 = man, 1 = woman); [2]**Risk** = self-reported chronic health condition that increases risk from Covid-19 (0 = no, 1 = maybe, 2 = yes); **CHB** = Current Health Behavior (SD = Social Distancing, H = Hygiene); **FHB** = Future Health Behavior (VB = Venturous Behavior); **M** = Public-health Message (SC = Self-centered, R = Responsible, Com = Compassionate, Av = Avoidant, S = Sociable); **MIP** = Mini-IPIP (A = Agreeableness, C = Conscientiousness, NE = Neuroticism, O = Openness, ST = Stability, PL = Plasticity, PF = General P Factor); **AMP** = Abbreviated Measure of Psychopathy (B = Boldness, M = Meanness, D = Disinhibition); **SD3** = Short Dark Triad (Nar = Narcissism, Mch = Machiavellianism, P = Psychopathy, DF = Dark Factor of Personality). **Note:** $p < .01$ = 1.0E-2, $p < .001$ = 1.0E-3.

Table S15
Descriptive Statistics for Personality Superfactors (N = *502)*

	Items	Scale	*M*	95% C.I.**	*Md*	*SD*	α
**Mini-IPIP***				bias-corrected			
Stability (C, A, rev. N)	12	0-4	2.82	[2.8 - 2.9]	2.8	0.62	.80
Plasticity (E, O)	8	0-4	2.19	[2.1 - 2.3]	2.1	0.79	.83
P (General Factor of Personality)	4	0-4	2.57	[2.5 - 2.6]	2.6	0.58	.85
**AMP***							
AMP Psychopathy[1]	33	0-3	0.64	[0.6 - 0.7]	0.58	0.41	.92
**SD3***							
D (Dark Factor of Personality)	27	0-4	1.34	[1.3 - 1.4]	1.30	0.57	.90

* Mini-IPIP: Mini – International Personality Item Pool; AMP: Abbreviated Measure of Psychopathy; SD3: Short Dark Triad.
** Bootstrapping estimates based on 1000 iterations.th

Table S16

Exploratory Analyses with Personality Superfactors and Health Behavior Endorsements: Partial Correlations Controlling for Gender, Age, and Risk Health Condition, One-tailed, with Bias-corrected Confidence Interval Estimates, N *= 502.*

**Personality superfactors**		**Current Health Behavior**		**Future Health Behavior**			**Carrier Scenario**	
		**Social distancing**	**Hygiene**	**Social distancing**	**Hygiene**	**Venturous behavior**	**Harmful behavior**	**Protecting others**
**MIP Stability**	r_p/p	.24 4.3E-08	.38 1.2E-18	.21 1.6E-06	.37 6.9E-18	-.09 2.1E-02	-.15 2.9E-04	.18 2.4E-05
98% b.c. C.I.		[.14 – .36]	[.27 – .48]	[.09 – .33]	[.26 – .48]	[-.24 – .03]	[-.24 – -.08]	[.08 – .28]
**MIP Plasticity**	r_p/p	.13 2.6E-03	.24 9.7E-08	.05 1.5E-01	.17 5.0E-05	.02 3.5E-01	-.08 4.0E-02	.15 3.5E-04
98% b.c. C.I.		[.03 – .25]	[.12 – .34]	[-.06 – .15]	[.06 – .29]	[-.09 – .13]	[-.15 – -.01]	[.05 – .25]
**MIP P Factor**	r_p/p	.23 3.2E-07	.37 1.6E-17	.16 2.4E-04	.33 2.9E-14	-.05 1.4E-01	-.14 8.8E-04	.20 4.7E-06
98% b.c. C.I.		[.12 – .34]	[.25 – .47]	[.05 – .28]	[.22 – .44]	[-.17 – .05]	[-.21 – -.07]	[.10 – .29]
**AMP Psychopathy**	r_p/p	-.15 9.2E-04	-.15 5.6E-04	-.21 1.3E-06	-.20 2.2E-06	.18 2.6E-05	.17 4.4E-05	-.17 4.5E-05
98% b.c. C.I.		[-.26 – -.03]	[-.28 – -.01]	[-.36 – -.06]	[-.35 – -.07]	[.03 – .33]	[.01 – .30]	[-.28 – -.08]
**SD3 Dark Factor**	r_p/p	-.14 1.5E-03	-.12 9.6E-03	-.13 2.5E-03	-.12 2.8E-03	.11 6.1E-03	.13 1.5E-03	-.10 1.3E-02
98% b.c. C.I.		[-.24 – -.04]	[-.23 – .01]	[-.27 – .01]	[-.26 – .01]	[-.04 – .29]	[.02 – .23]	[-.20 – .00]

Note: Controlling for sex, age, and risk health condition.

Note: Bootstrap estimation of C.I. with 1000 iterations. **Note:** $p < .01$ = 1.0E-2, $p < .001$ = 1.0E-3.

Note: MIP = Mini-IPIP (Stability subsumes Conscientiousness, Agreeableness, and low Neuroticism; Plasticity subsumes Extraversion and Openness; and the General P Factor subsumes all five factors with Neuroticism reversed); AMP = Abbreviated Measure of Psychopathy (AMP Psychopathy subsumes Boldness, Meanness and Disinhibition); SD3 = Short Dark Triad (the SD3 Dark Factor subsumes Narcissism, Machiavellianism, and Psychopathy).

Table S17

Exploratory Analyses with Personality Superfactors and Public-health Message Appeal: Partial Correlations Controlling for Gender, Age, and Risk Health Condition, One-tailed, with Bias-corrected Confidence Interval Estimates, N = *502.*

Personality dimensions		***Public-health Message Appeal***									
		**Self-centered**		**Responsible**		**Compassionate**		***Avoidant***		**Sociable**	
**MIP Stability**	r_p/p	0.27	7.1E-10	.26	1.8E-09	.31	7.5E-13	.23	1.8E-07	.19	6.9E-06
98% b.c. C.I.		[0.15	– .37]	[.14	– .36]	[.21	– .40]	[.11	– .32]	[.07	– .32]
**MIP Plasticity**	r_p/p	0.17	9.7E-05	.17	5.1E-05	.15	2.6E-04	.14	1.1E-03	.16	1.8E-04
98% b.c. C.I.		[0.05	– .27]	[.06	– .28]	[.05	– .25]	[.02	– .24]	[.05	– .26]
**MIP P Factor**	r_p/p	0.26	2.2E-09	.26	2.3E-09	.28	9.9E-11	.22	4.9E-07	.21	1.3E-06
98% b.c. C.I.		[0.14	– .37]	[.14	– .36]	[.17	– .37]	[.10	– .32]	[.09	– .32]
**AMP Total**	r_p/p	-0.08	3.2E-02	-.11	8.3E-03	-.25	7.1E-09	-.08	3.5E-02	-.06	1.0E-01
98% b.c. C.I.		[-0.18	– .03]	[-.21	– .01]	[-.36	– -.14]	[-.18	– .01]	[-.15	– .03]
**SD3 Total**	r_p/p	-0.06	8.9E-02	-.09	2.8E-02	-.21	1.1E-06	-.01	3.9E-01	.01	4.6E-01
98% b.c. C.I.		[-0.17	– .04]	[-.19	– .02]	[-.31	– -.11]	[-.11	– .08]	[-.10	– .10]

Note: Controlling for sex, age, and risk health condition.

Note: Bootstrap estimation of C.I. with 1000 iterations. **Note:** $p < .01$ = 1.0E-2, $p < .001$ = 1.0E-3.

Note: MIP = Mini-IPIP (Stability subsumes Conscientiousness, Agreeableness, and low Neuroticism; Plasticity subsumes Extraversion and Openness; and the General P Factor subsumes all five factors with Neuroticism reversed); AMP = Abbreviated Measure of Psychopathy (AMP Psychopathy subsumes Boldness, Meanness and Disinhibition); SD3 = Short Dark Triad (the SD3 Dark Factor subsumes Narcissism, Machiavellianism, and Psychopathy).

Table S18

Exploratory Multiple Regression Analyses Predicting Message Appeal from Personality Variables (N = *502)*

**Personality dimensions**	***Public-health Message Appeal***									
	**Self-centered**		**Responsible**		**Compassionate**		***Avoidant***		**Sociable**	
**Model 1**	**β**	***p***	**β**	***p***	**β**	***p***	**β**	***p***	**β**	***p***
**MIP Agreeableness**	0.25	<.0001	0.29	<.0001	0.39	<.0001	0.25	<.0001	0.21	<.0001
**MIP Conscientiousness**	0.10	.0260	0.07	.1450	0.08	.0615	0.07	.1068	0.01	.7450
**MIP Neuroticism**	0.04	.3601	0.04	.3739	-0.05	.2462	0.02	.6232	0.11	.0245
**MIP Extraversion**	-0.04	.4034	-0.02	.5994	-0.04	.3994	-0.02	.7271	-0.02	.7462
**MIP Openness**	-0.02	.6758	-0.02	.7047	0.01	.8121	-0.01	.7732	-0.05	.3364
**Model 2**	**β**	***p***	**β**	***p***	**β**	***p***	**β**	***p***	**β**	***p***
**AMP Boldness**	0.05	.3095	0.05	.3260	0.03	.6099	0.01	.8029	0.05	.3379
**AMP Meanness**	-0.15	.0087	-0.23	.0001	-0.31	<.0001	-0.12	.0352	-0.19	.0015
**AMP Disinhibition**	-0.08	.1853	-0.04	.5173	-0.10	.0817	-0.08	.2387	0.00	.9536
**Model 3**	**β**	***p***	**β**	***p***	**β**	***p***	**β**	***p***	**β**	***p***
**SD3 Narcissism**	0.15	.0019	0.16	.0011	0.08	.0945	0.13	.0080	0.16	.0010
**SD3 Machiavellianism**	-0.10	.0691	-0.18	.0015	-0.16	.0025	0.00	.9611	-0.06	.2996
**SD3 Psychopathy**	-0.20	.0005	-0.16	.0043	-0.27	<.0001	-0.24	<.0001	-0.16	.0064

Note: MIP: Mini-IPIP; AMP: Abbreviated Measure of Psychopathy; SD3: Short Dark Triad.

Figure 2S

Conscientiousness and neuroticism interact in predicting self-reported social distancing (corrected for age, gender identity, and the presence of a chronic health condition that increases the risk of Covid-19), $N = 502$.

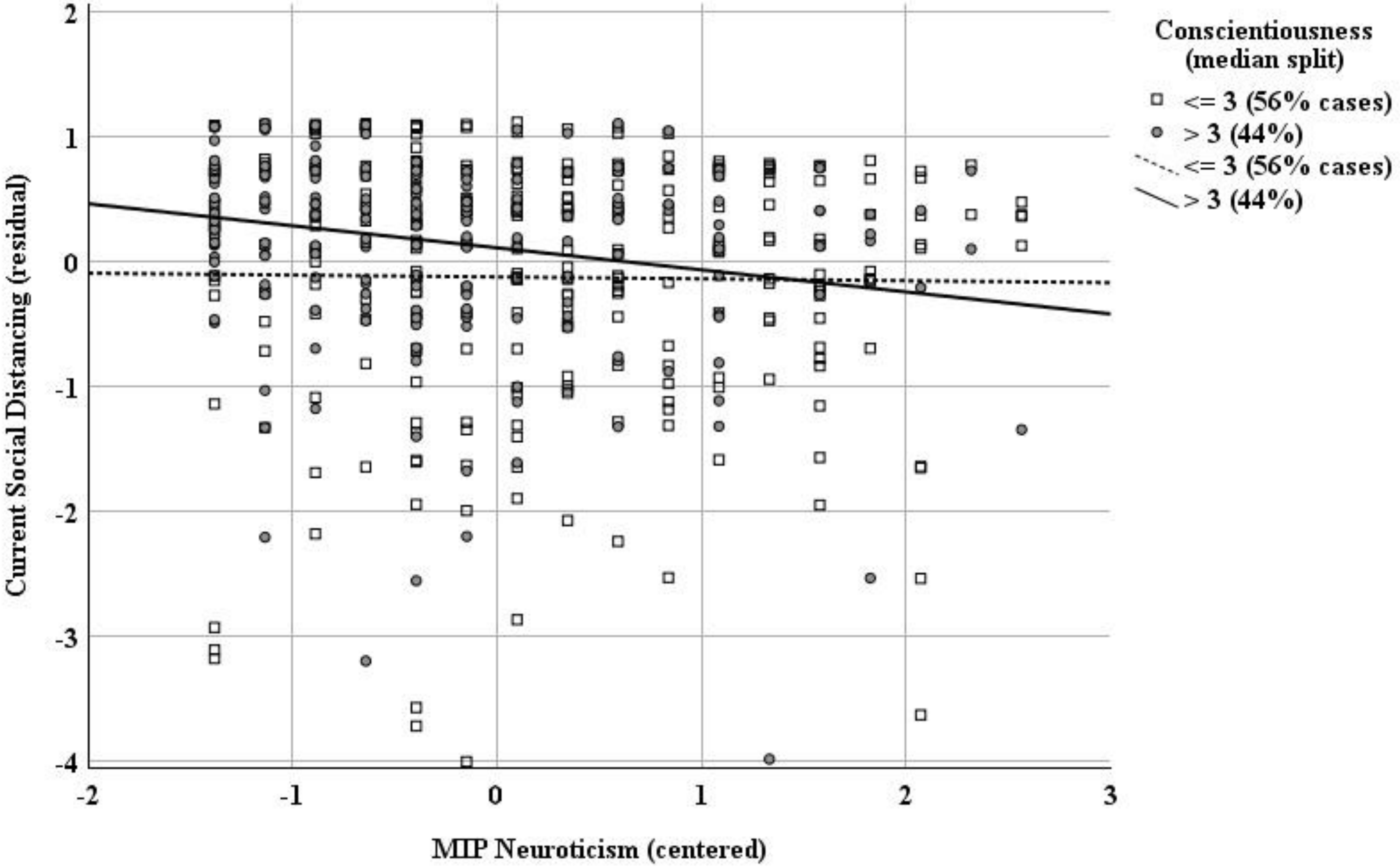

Figure S3

Conscientiousness and agreeableness as they relate to self-reported intent for future social-distancing (corrected for age, gender identity, and the presence of a chronic health condition that increases the risk of Covid-19), *N* = 502. (Note that the test of interaction was significant in null-hypothesis testing but not in a bootstrap analysis.)

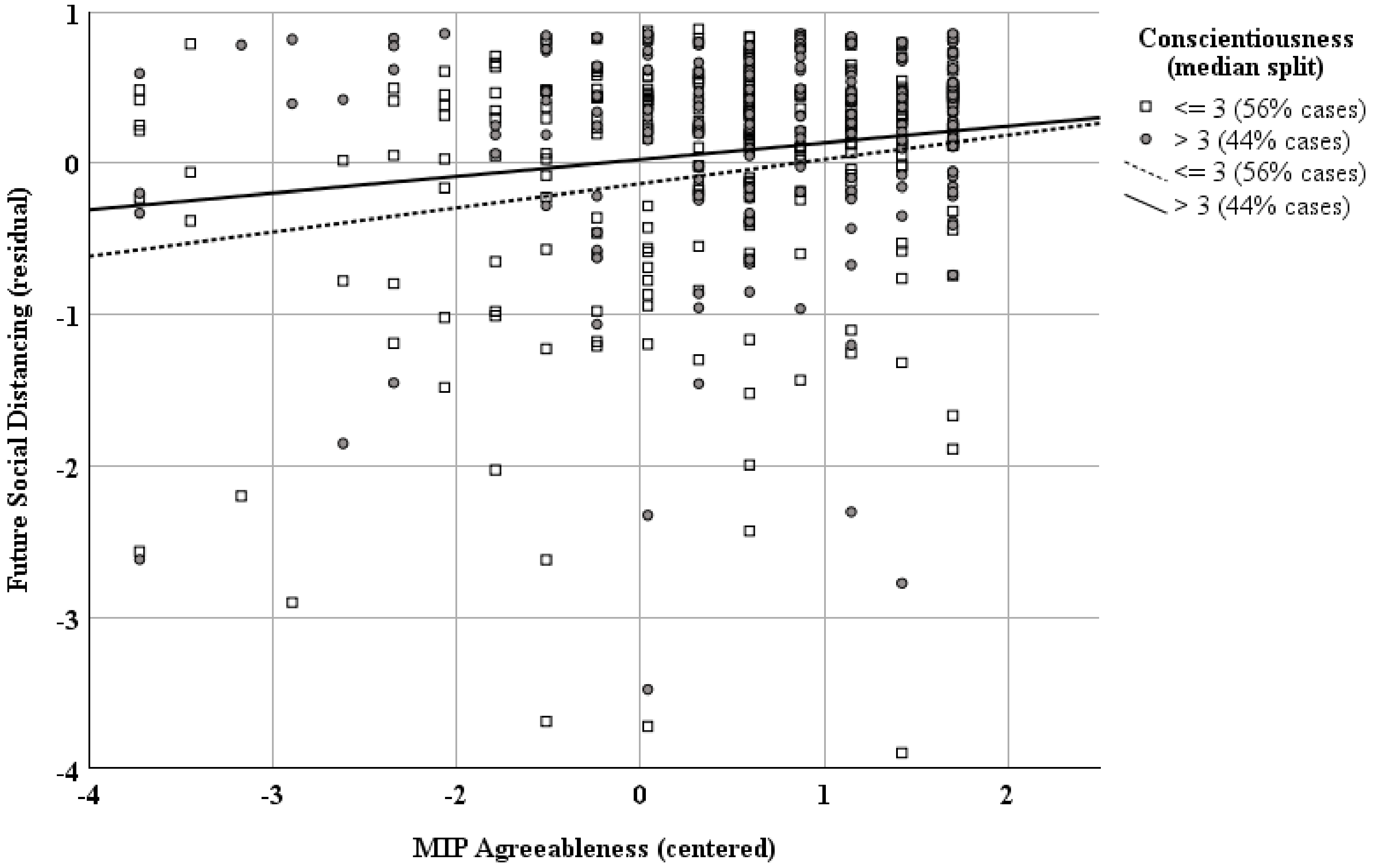

Tale S19

Unplanned Multiple Regression Analyses Predicting Key Health-related Variables from Normal-range Personality Dimensions (N = 502)

Model	Step 2[1]	Predictors	*R*	*Adj. R²*	*p*	β	*p*	*b*	95% b.c. C.I.	VIF	DW
1	**Current Health Behavior: Social Distancing**	Agreeableness	.35†	.108†	5.3E-11	0.17†	8.0E-4	0.11	0.02 – 0.18	1.35	2.04
		Conscientiousness				0.13*	4.4E-3	0.09	0.02 – 0.16	1.13	
		Neuroticism				-0.05	3.5E-1	-0.03	-0.08 – 0.03	1.31	
		Extraversion				0.02	6.8E-1	0.01	-0.04 – 0.06	1.32	
		Openness				0.02	7.4E-1	0.01	-0.05 – 0.07	1.23	
2	**Future Health Behavior: Social Distancing**	Agreeableness	.31†	.097†	3.7E-8	0.15*	2.0E-3	0.14	0.04 – 0.24	1.35	1.99
		Conscientiousness				0.11^	1.7E-2	0.11	0.01 – 0.22	1.13	
		Neuroticism				-0.07	1.4E-1	-0.06	-0.16 – 0.02	1.31	
		Extraversion				0.00	1.0E+0	0.00	-0.06 – 0.07	1.32	
		Openness				-0.08	1.1E-1	-0.07	-0.16 – 0.01	1.23	
3	**Future Health Behavior: Hygiene**	Agreeableness	.44†	.181†	1.8E-19	0.28†	6.0E-9	0.27	0.17 – 0.39	1.35	2.01
		Conscientiousness				0.15*	6.1E-4	0.16	0.06 – 0.27	1.13	
		Neuroticism				-0.12^	1.3E-2	-0.10	-0.18 – -0.03	1.31	
		Extraversion				0.04	3.6E-1	0.03	-0.03 – 0.10	1.32	
		Openness				-0.05	2.3E-1	-0.06	-0.14 – 0.03	1.23	
4	**Carrier Scenario: Harmful Behavior**	Agreeableness	.25*	.05*	8.7E-5	-0.15*	2.4E-3	-0.02	-0.05 – 0.00	1.35	2.06
		Conscientiousness				-0.02	5.9E-1	0.00	-0.02 – 0.01	1.13	
		Neuroticism				0.06	2.2E-1	0.01	0.00 – 0.02	1.31	
		Extraversion				0.03	5.9E-1	0.00	-0.01 – 0.03	1.32	
		Openness				-0.02	7.1E-1	0.00	-0.04 – 0.02	1.23	

[1]Each model controls for age, gender identity, and health risk status in a previous step omitted for brevity. Bootstrap estimation of bias-corrected C.I. with 1000 iterations. VIF: variance inflation factors. DW: Durbin-Watson statistic.

^ $p < .05$ = 5.0E-2; * $p < .01$ = 1.0E-2; † $p < .001$ = 1.0E-3

Table S20

Unplanned Multiple Regression Analyses Predicting Health-related and Harmful Behavior Endorsement from the Triarchic Model of Psychopathy Dimensions (N = *502).*

Model	Step 2[1]	Predictors	*R*	*Adj. R^2*	*p*	β	*p*	*b*	95% b.c. C.I.	VIF	DW
1	**Current**	Boldness	.31†	.087†	3.1E-09	0.08	1.4E-01	0.08	-0.03 – 0.17	1.50	2.01
	**Health Behavior:**	Meanness				-0.10	7.2E-02	-0.13	-0.33 – 0.06	1.80	
	**Social Distancing**	Disinhibition				-0.15^	1.7E-02	-0.19	-0.35 - -0.04	2.10	
2	**Future**	Boldness	.32†	.094†	4.7E-10	0.04	4.8E-01	0.05	-0.10 – 0.20	1.50	1.97
	**Health Behavior:**	Meanness				-0.10	7.7E-02	-0.18	-0.40 – 0.04	1.80	
	**Social Distancing**	Disinhibition				-0.19*	2.7E-03	-0.34	-0.60 - -0.09	2.10	
3	**Future**	Boldness	.39†	.140†	2.2E-15	0.17†	9.2E-04	0.27	0.10 - 0.43	1.50	1.96
	**Health Behavior:**	Meanness				-0.21†	2.2E-04	-0.39	-0.66 - -0.14	1.80	
	**Hygiene**	Disinhibition				-0.20†	8.0E-04	-0.40	-0.65 - -0.14	2.10	
4	**Carrier**	Boldness	.29†	.070†	1.9E-7	-0.06	2.2E-01	-0.02	-0.04 – 0.01	1.50	2.05
	**Scenario:**	Meanness				0.15^	1.0E-02	0.04	0.01 – 0.09	1.80	
	**Harmful Behavior**	Disinhibition				0.13^	3.9E-02	0.04	0.00 – 0.09	2.10	
5	**Future**	Boldness	.23†	.040†	2.1E-4	0.01	8.9E-01	0.01	-0.14 – 0.16	1.50	2.04
	**Health Behavior:**	Meanness				-0.01	8.7E-01	-0.02	-0.27 – 0.20	1.80	
	**Venturous**	Disinhibition				0.22†	6.3E-04	0.38	0.10 – 0.68	2.10	

[1]Each model controls for age, gender identity, and health risk status in a previous step omitted for brevity. Bootstrap estimation of bias-corrected C.I. with 1000 iterations. VIF: variance inflation factors. DW: Durbin-Watson statistic.

^ $p < .05$ = 5.0E-2; * $p < .01$ = 1.0E-2; † $p < .001$ = 1.0E-3.

Table S21

*Unplanned Multiple Regression Analyses Predicting Health- related and Harmful Behavior Endorsement from the Dark Triad of Personality Dimensions (*N *= 502)*

Model	Step 2[1]	Predictors	*R*	*Adj. R²*	*p*	β	*p*	*b*	95% b.c. C.I.	VIF	DW
1	**Current**	Narcissism	.39†	.118†	1.0E-12	0.14*	2.6E-03	0.11	0.08 – 0.19	1.26	2.03
	**Health Behavior:**	Machiavellianism				-0.07	2.0E-01	-0.05	-0.14 – 0.03	1.70	
	**Social Distancing**	Psychopathy				-0.27†	4.9E-06	-0.25	-0.37 – -0.13	1.95	
2	**Future**	Narcissism	.31†	.085†	4.6E-9	0.10^	3.8E-02	0.11	0.01 – 0.21	1.26	1.97
	**Health Behavior:**	Machiavellianism				-0.03	5.5E-01	-0.04	-0.14 – 0.06	1.70	
	**Social Distancing**	Psychopathy				-0.24†	5.5E-05	-0.32	-0.51 – -0.13	1.95	
3	**Future**	Narcissism	.35†	.121†	6.8E-12	0.18†	2.1E-04	0.20	0.10 – 0.32	1.26	1.94
	**Health Behavior:**	Machiavellianism				-0.12^	3.6E-02	-0.14	-0.27 – 0.00	1.70	
	**Hygiene**	Psychopathy				-0.23†	9.2E-05	-0.33	-0.52 - -0.15	1.95	
4	**Carrier**	Narcissism	.25†	.052†	1.3E-5	-0.02	6.5E-01	0.00	-0.02 – 0.01	1.26	2.06
	**Scenario:**	Machiavellianism				0.00	9.3E-01	0.00	-0.2 – 0.03	1.70	
	**Harmful Behavior**	Psychopathy				0.20†	8.9E-04	0.05	0.01 – 0.08	1.95	
5	**Future**	Narcissism	.20*	.028*	2.6E-3	-0.03	5.0E-01	-0.03	-0.13 – 0.06	1.26	2.05
	**Health Behavior:**	Machiavellianism				-0.03	5.9E-01	-0.03	-0.15 – 0.09	1.70	
	**Venturous**	Psychopathy				0.23†	2.5E-04	0.29	0.10 – 0.48	1.95	

[1] Each model controls for age, gender identity, and health risk status in a previous step omitted for brevity. Bootstrap estimation of bias-corrected C.I. with 1000 iterations. VIF: variance inflation factors. DW: Durbin-Watson statistic.

^ $p < .05 = 5.0E-2$; * $p < .01 = 1.0E-2$; † $p < .001 = 1.0E-3$.

Appendix SA

Public-health Messages

Self-centered:

"Keep yourself healthy!
First and foremost, prioritize your own health. Control other people's distance from you, and keep them at least 6 feet away. Use soap and hand sanitizer often to keep other people's germs away. Leave your home only to take care of your basic needs (food and medical care). If possible, work from home."

Responsible:

"Take personal responsibility!
You have an obligation to safeguard your health. Be mindful how far you are from other people, and maintain a distance of at least 6 feet. Carefully wash your hands with soap, or rub them with hand sanitizer thoroughly. You should not leave your home, except for such necessities as food or medical care. If possible, work from home."

Compassionate:

"Help protect the vulnerable!
Consider the well-being of people who are older or have chronic health conditions. To reduce their and everyone's risk, try to keep a distance of at least 6 feet away from others. Help slow the spread of germs by washing your hands with soap and using hand sanitizer often. To protect my community, only leave the house if you need necessities, like food, medication, or to help a person in need. If possible, work from home."

Avoidant:

"Avoid the disease!
It is essential to stay clear of the contagion. To remain disease-free, steer away from people by keeping a distance of at least 6 feet. To ward off germs, cleanse your hands thoroughly with soap or hand sanitizer often. Avoid leaving your home, except to get food or medical care. If possible, work from home."

Sociable:

“Distant socializing, not social distancing!
You don't have to give up on the fun of socializing because of the coronavirus. You have to keep 6 feet a part in person, but you can meet up online or chat over the phone one-on-one or in a group session. That way, you can leave your home only for necessities, like getting food or medical care. And if you do, don't spoil the party - wash your hands with soap or use hand sanitizer often. If possible, work from home.”
